# Network-based prediction of anti-cancer drug combinations

**DOI:** 10.3389/fphar.2024.1418902

**Published:** 2024-08-28

**Authors:** Jue Jiang, Xuxu Wei, YuKang Lu, Simin Li, Xue Xu

**Affiliations:** ^1^ School of Medicine, Wuhan University of Science and Technology, Wuhan, Hubei, China; ^2^ Key Laboratory of Chinese Internal Medicine of Ministry of Education, Dongzhimen Hospital, Beijing University of Chinese Medicine, Beijing, China

**Keywords:** cancer, drug combination, protein-protein interaction network, network proximity, community detection

## Abstract

Drug combinations have emerged as a promising therapeutic approach in cancer treatment, aimed at overcoming drug resistance and improving the efficacy of monotherapy regimens. However, identifying effective drug combinations has traditionally been time-consuming and often dependent on chance discoveries. Therefore, there is an urgent need to explore alternative strategies to support experimental research. In this study, we propose network-based prediction models to identify potential drug combinations for 11 types of cancer. Our approach involves extracting 55,299 associations from literature and constructing human protein interactomes for each cancer type. To predict drug combinations, we measure the proximity of drug-drug relationships within the network and employ a correlation clustering framework to detect functional communities. Finally, we identify 61,754 drug combinations. Furthermore, we analyze the network configurations specific to different cancer types and identify 30 key genes and 21 pathways. The performance of these models is subsequently assessed through *in vitro* assays, which exhibit a significant level of agreement. These findings represent a valuable contribution to the development of network-based drug combination design strategies, presenting potential solutions to overcome drug resistance and enhance cancer treatment outcomes.

## 1 Introduction

There has been huge progress in the discovery of drug combination therapies for cancer treatment in recent years, regarding the advantages of better efficacy, lower dose requirement, and fewer adverse side effects (Zhou et al., 2019; Jin et al., 2020; Zhang et al., 2020). Despite the accelerating clinical impact and a substantial increase in the number of FDA-approved drug combinations from 2011 to 2021 (Fudio et al., 2022). The current speed of development of drug combinations still falls short of meeting the urgent clinical needs of cancer patients.

Generally, there were two types of strategies to foster the development of drug combinations: computational-based methods and wet experiment. Considering the vast size of chemical space (comprising >10^60^ molecules), AI technologies such as deep learning, machine learning, and system biology have been widely applied to increase success rates in drug development (Güvenç Paltun et al., 2021; Paul et al., 2021). For instance, DeepSynergy implemented a feed-forward neural network by utilizing chemical descriptors for drug A, drug B, and genomic information from cell lines to predict anti-cancer drug synergy (Preuer et al., 2018). Similarly, MatchMaker (Kuru et al., 2022) and AuDNNsynergy (Zhang et al., 2021) automatically extracted features from drug structures and genomic information of cell lines for drug combination prediction. Machine learning methods encompassed supervised learning, unsupervised learning, and semi-supervised learning approaches to tackle drug combination prediction tasks. DECREASE incorporated a compendium of 23,595 drug combination matrices tested across various cancer cell lines to predict the effects of drug combinations (Ianevski et al., 2019). ComboFM employed two molecular fingerprints of drugs, concentration values of both drugs, and gene expression profiles of cancer cell lines as input features, utilizing a factorization machine model to predict complete dose-response matrices (Julkunen et al., 2020). Although the models achieved relatively high predictive performance in the prediction of drug combinations, almost all the studies circumvented the problem of lack of target information, which probably oversimplified the actual situation of the drug combination in cancer treatment. To address the problem, Cheng et al. designed a network-based proximity measure between targets of two drugs for predicting drug combinations. This approach provided a robust network methodology for identifying effective combination therapies (Cheng et al., 2019). However, the study ignored the heterogeneity of diseases and missed multiple PPIs in literature-derived low-throughput experiments.

Today, high-throughput screening (HTS) assays, using biochemical, binding-based, and cell-based approaches, automatically mined compound libraries for biologically active molecules, and thus have opened new avenues of drug discovery in pharmacological industries (Blay et al., 2020; van Vlijmen et al., 2021). In a study focusing on diffuse midline glioma (DMG), Lin et al. (2019) conducted sequential quantitative HTS using six DMG cultures and a library of 2,706 approved and investigational drugs, generating 19,936 individual dose-response profiles for single-agent compounds. Similarly, Di Marco et al. (2020) utilized an HTS assay with a library of 44,000 non-proprietary compounds to identify two compounds that effectively reduced mitochondrial calcium influx. However, HTS success rates are only roughly 50% successful obtention of a hit. The main miscalculations stem from poorly validated targets, limited screening libraries, and artificial assay systems that do not closely resemble physiological conditions (Costa et al., 2020). Consequently, the selection of drug combinations still remains far from optimal. HTS alone cannot determine all possible plausible combinations among drugs.

In our research, we aimed to predict small-molecule combinations for cancer treatment by considering the actual complexities of drug combinations and accounting for heterogeneity within cancer cell lines. To achieve this, we extracted valuable associations from 14,394 PubMed literature articles, encompassing information on drugs, genes, gene regulation, cancer cell types, and cancer types. Then, human protein interactomes were separately constructed for each cancer phenotype by integrating drug-protein interaction information based on our collected literatures and DrugBank database. We designed a network-based strategy for drug combination prediction by measuring the network proximity of drug–drug relationships and employing a correlation clustering framework for community detection. This approach represents a significant advancement in predicting effective drug combinations for cancer treatment.

## 2 Materials and methods

### 2.1 Drug-gene network construction

55,299 cancer-related literatures ranging from 1995 to 2021 was collected from PubMed. Review, meta-analysis, clinical assays, and computational studies were screened out. Keywords about drug, gene, cancer cell line, cancer type, and drug treatment outcome were extracted from the abstracts of remaining 14,394 literatures by text-mining, with the aim to construct semantic relationship among the keywords, i.e., drug X leads to certain treatment outcome on a specific cancer cell line from a specific type of cancer by regulating certain genes. To correct the noisy information and improve the robustness, three medical experts and 10 medical students manually curated the relationships. Drug-target relationships were further downloaded from the DrugBank database and were then integrated with our manually curated relationships.

A large gene-gene network was constructed, as shown in Algorithm 1. The network edge is the connection between two genes. For each type of cancer (non-small cell lung cancer, colon cancer, acute myeloid leukemia, pancreatic cancer, breast cancer, gastric cancer, ovarian cancer, renal cancer, liver cancer, head and neck cancer, prostate cancer, cervical cancer, endometrial cancer, thyroid cancer), a cancer-specific network was constructed, respectively.

Algorithm 1. Algorithm for cancer-specific network construction.

Input: cancer-drug-gene relation matrix ${DGR}^{n\times m}$ and drug-gene information list $List_{DG}$ from collected references.
Output: cancer-specific network $G_{c}<V_{c},E_{c}>$ .
Steps:
1. For each type of cancer, iterate the drug-gene information list and search for related genes that appeared in the same literature.
2. Edges are created among those genes in the same literature.
3. Delete isolated nodes and edge loops.
4. Return nodes set and edge set.

### 2.2 Community detection in cancer-specific networks

Louvain community detection algorithm consisted of two main phases, i.e., local maxima modularity and new network building. The changed modularity of a node and community *C* was calculated as follows.

$$\Delta Q=\frac{1}{2m}\left\{\left[\left(C_{in}+k_{i}^{in}\right)-\frac{{\left(C_{out}+k_{i}\right)}^{2}}{2m}\right]-\left[C_{in}-\frac{{\left(C_{out}\right)}^{2}}{2m}-\frac{k_{i}^{2}}{2m}\right]\right\}$$

where $C_{in}$ represents the sum weights of inner links. $C_{out}$ means the sum weights of outer links. $k_{i}^{in}$ represents the inner degree of node *i*. $k_{i}$ represents the total degree of node *i*.

Algorithm 2. Louvain community detection algorithm.

Input: Cancer-specific networks $G_{ca}<V_{ca},E_{ca}>$ .
Output: Communities $C=\left\{C_{1},C_{2},\ldots ,C_{n}\right\}$ .
Steps:
1. Initialize each node as a single community.
2. Search neighbor nodes of node *i*. Calculate, if ΔQ >0, then add the neighbor node *i* into the corresponding community *C*.
3. Repeat the process until the scale of communities does not change.
4. Construct a new graph to compress nodes in the same community as a node. And change links and weights according to the changed nodes.
5. Repeat Steps 1–3 until the nodes do not change.

### 2.3 Network-based proximity measure

Network-based proximity used the separation measure to quantify the network-based relationship between the targets of two drugs. The separation measure scoring algorithm consisted of two steps, i.e., the searching step and the calculation step. The detail of the separation measure scoring algorithm was shown below.

Algorithm 3. Algorithm for separation measure scoring.

Input: Gene sets *genes_a* and *genes_b*.
Output: Separation measure of *genes_a* and *genes_b*, $s_{AB}$ .
Steps:
1. Search target genes modules *A* of *drug_a* and *B* of *drug_b*.
2. Calculate the mean shortest distance within modules *A* and *B* which are noted as ${\bar{d}}_{AA}$ and ${\bar{d}}_{BB}$ .
3. Calculate the mean shortest distance ${\bar{d}}_{AB}$ within links between modules *A* and *B*.
4. Calculate the separation measure score $s_{AB}$ as follows. $s_{AB}={\bar{d}}_{AB}-\frac{{\bar{d}}_{AA}+{\bar{d}}_{AB}}{2}$ .

### 2.4 Drug combination prediction in cancer-specific network

We predicted drug combination by combining network-based proximity and communities in the cancer-specific network. For a drug pair, A and B have targets $T_{A}\text{ and }T_{B}$ . Network-based proximity between the targets of the drug pair, $S_{AB}$ , was calculated by Algorithm 3.

Furthermore, we defined a cancer-specific network as $G_{ca}<V_{ca},E_{ca}>$ . We detected the communities within $G_{ca}$ by Algorithm 2, and separated $G_{ca}$ into $C=\left\{C_{1},C_{2},...,C_{i}\right\}$ . For a drug X, we calculated the number and the ratio of drug targets within each community to quantify whether the drug X hits a community:

$$“C_{X}=\left\{C_{j}│\left|C_{j}\cap T_{X}\right|\geq \min\left(\alpha ,\beta \left|C_{j}\right|\right),\left|C_{j}\right|\geq \gamma ,\;C_{j}\in C\right\}$$

Where $C_{X}$ represents the community hit by the drug X, $C_{j}$ is the community within $G_{ca}$ , $T_{X}$ is the target set hit by the drug X. α (greater than 0) represents the number of targets within the community that the drug must hit, while β (between 0 and 1) representing the proportion of drug targets that must be hit for the community to be considered as hit. γ is a constant greater than or equal to 1, representing the minimum number of targets that the community *Cj* should contain to calculate community hits. This parameter is used to prevent false positive results. α , β , and γ were set to 10, 0.15, and 10 based on community size, i.e., in a community with more than 10 nodes, drug X was identified to hit the community when the drug hit 10 or 15% nodes. If there is community overlap hit by drug pair (A, B), i.e., $C_{A}\cap C_{B}\neq \emptyset$ , $S_{AB}<0$ , we define the drug pair (A, B) has drug combination.

### 2.5 Materials

Bortezomib (S1013, batch No. 17), Curcumin (S1848, batch No. 07), Palbociclib HCl (S1116, batch No. 14), Dihydroartemisinin (S2290, batch No. 09) and Sorafenib (S7397, batch No. 07) was purchased from Selleck Chemicals LLC (United States). All human cancer cells were purchased from Procell Life Science&Technology Co., Ltd. (China). SW480, HCT-116 and BxPC-3 cells were maintained in RPMI-1640 (GIBCO, United States) containing 10% fetal bovine serum (FBS; GIBCO, United States) and 1% penicillin/streptomycin (GIBCO, United States). MCF-7 was maintained in Dulbecco’s Modified Eagle’s medium (DMEM; GIBCO, United States) containing 10% FBS, 10 μg/mL insulin (MERCK, United States), and 1% penicillin/streptomycin. MDA-MB-231 and Panc-1 cells were maintained in DMEM containing 10% FBS and 1% penicillin/streptomycin. HL-60 and MV-4-11 cells were maintained in Iscove’s Modified Dulbecco Medium (IMDM; GIBCO, United States) containing 20% FBS and 1% penicillin/streptomycin. All cell lines were authenticated with short tandem repeats (STR) analysis, cultured for fewer than 6 months after resuscitation, and tested for *mycoplasma* contamination every 3 months using MycoAlert (Lonza). Annexin V-FITC Apoptosis Kit was purchased from ZOMANBIO (China).

### 2.6 Inhibitors treatments

HCT-116 and SW480 cell lines were treated with a fixed BTZ concentration of 30 nM and 80 nM, respectively, alongside increasing concentrations of Curcumin (5 μM–60 μM). MCF-7 and MDA-MB-231 cell lines were exposed to a fixed PD concentration of 10 μM and 5 μM, respectively, with escalating doses of Curcumin (5 μM–60 μM for MCF-7 and 2.5 μM–40 μM for MDA-MB-231). Panc-1 cell line received a fixed DHA concentration of 10 μM with incremental additions of Sorafenib (1 μM–40 μM). BxPC-3 cell line was treated with a fixed DHA concentration of 60 μM and various Sorafenib concentrations (2.5 μM–20 μM). MV-4-11 cell line was subjected to a fixed BTZ concentration of 5 nM with a range of DHA concentrations (50 nM–600 nM). HL-60 cell line was treated with a fixed DHA concentration of 500 nM alongside escalating BTZ concentrations (1.25 nM–10 nM). The 48-h treatment duration was chosen to ensure adequate exposure and interaction time between the drugs. After this period, the Combination Index (CI) values were determined for each combination to assess the nature of drug interactions, with interpretations as follows: CI > 1 suggests antagonism, CI = 1 indicates an additive effect, 0.7 < CI < 1 indicates slight synergism, 0.3 < CI < 0.7 indicates synergism, and CI < 0.3 indicates strong synergism. The average CI values for each cell line were calculated to provide a quantitative measure of the overall drug interaction effects.

### 2.7 Cell death assay and flow cytometry analysis

Cell death assay was performed using the Annexin V-FITC Apoptosis Kit according to the manual. Briefly, 5 × 10^5^ cells were resuspended in 500 μL binding buffer with 5 μL Annexin V-FITC and 10 μL PI staining solution for 10 min at room temperature. The acquisition was performed on the Accuri C6 (BD Biosciences, Franklin Lakes, NJ, United States) and data were analyzed with FlowJo software (Tree Star, Ashland, OR, United States).

### 2.8 Statistic analysis

Statistical analyses and data visualization were conducted utilizing SPSS (V26.0), GraphPad (V9.5) or R software (V4.2.3). To assess the statistical significance of differences between groups, a one-way ANOVA was applied, followed by Tukey’s *post hoc* test for multiple comparisons. The *p* values obtained from these analyses were used to determine the significance of the differences, with *p* < 0.05 considered to indicate statistical significance. All cellular experiments were conducted with a minimum of three biological replicates.

Five different statistic methods were used to estimate performance of the network-based strategy for drug combination prediction, including Exact, Wilson, Agresti, Clopper-Pearsons, and Jeffreys.

## 3 Results

### 3.1 Network-based proximity measure and community detection of drug-drug relationships

**FIGURE 1:**
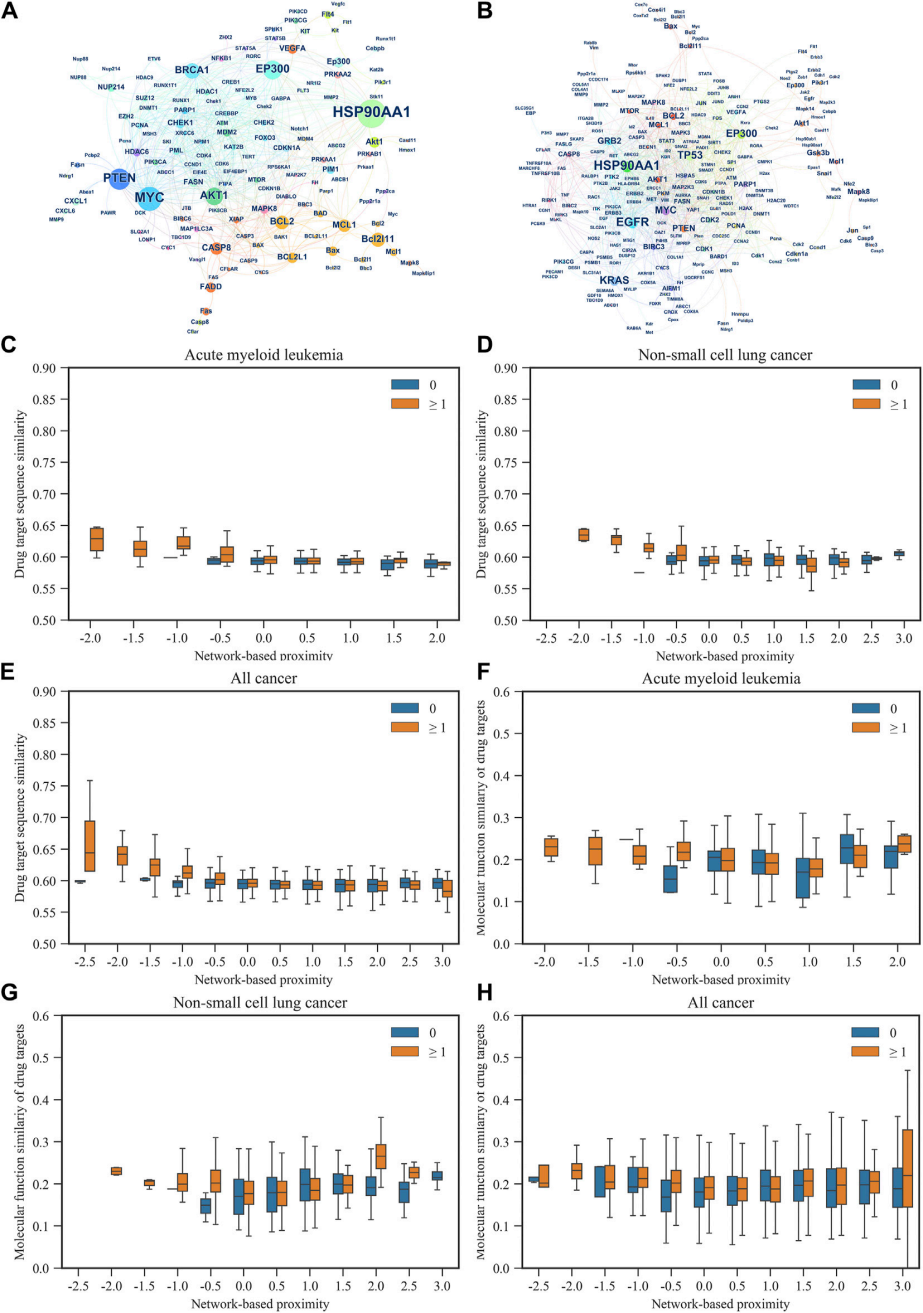
The relationship between gene networks and gene sequence similarity of AML and NSCLC, as well as network-based proximity networks. **(A,B)** respectively illustrate the gene networks of AML and NSCLC. Each node represents a gene, and the size of the node and its label indicate the importance of the gene in the network (represented by betweenness centrality). The color of the nodes represents the network communities to which the genes belong. The edges between two nodes represent potential interactions between genes under certain mechanisms. **(C–E)** respectively demonstrate the relationship between gene sequence similarity and network-based proximity in AML, NSCLC, and all cancer types included in this study. **(F–H)** respectively illustrate the relationship between gene functional similarity and network-based proximity in AML, NSCLC, and all cancer types included in this study.

We first collected cancer-related literatures dated from 1995 to the 2020 in PubMed. Keywords including drug, gene, cancer cell line, cancer type and drug treatment outcome were extracted from each abstract, and sematic associations among 6,261 drugs, 3,764 genes, 2,002 cancer cell lines, and 73 cancer types were identified. After standardization of drugs, genes, cancer cell lines, and cancer types based on PubChem, DrugBank, and UniProt, associations between 1,317 drugs and 1,315 genes were obtained. Next, background protein–protein interactions (PPIs) were assembled from five data sources, i.e., BioGRID (https://thebiogrid.org/), MINT (https://mint.bio.uniroma2.it/), BIND (http://bind.ca), DIP (http://dip.doe-mbi.ucla.edu), IntAct (https://www.ebi.ac.uk/intact/) and HPRD (http://hprd.org/index_html), which included 27,123 nodes and 663,114 edges. Based on the PubMed-derived drug-gene associations and the background network, we constructed networks for 11 most common cancer subtypes, including non-small cell lung cancer (NSCLC), colon cancer, acute myeloid leukemia (AML), pancreatic cancer, breast cancer, ovarian cancer, liver cancer, prostate cancer, osteosarcoma, glioma (Figures 1A, B; Supplementary Figures S1–S9).

For each cancer subtype, network-based proximity of drug–drug relationships were measured by comparing the mean shortest distance within the interactome between the targets of each of two drugs, to the mean shortest distance between the target pairs of two drugs (Cheng et al., 2019). For network-based proximity <0, the two drugs were denoted as a drug combination, while for network-based proximity ≥0, the two drugs were denoted as no interaction. We further examined associations of the network-based proximity with molecular function similarity and sequence similarity of target proteins. As shown in Figures 1C–E, sequence similarity of target proteins decreased with the increase of network-based proximity. Also, we found that molecular function similarity of target proteins derived from Gene Ontology annotations decreased with the increase of network-based proximity (Figures 1F–H). The same results were also found in the other cancer types (Supplementary Figures S10, S11). These results indicated that for the two drug-target modules with separated topology (network-based proximity ≥0), the drugs were pharmacologically distinct, while for the two drug-target modules with overlapping topology (network-based proximity <0), the drugs had higher similarities in their biological and functional profiles.

In addition, we used the Louvain method for community detection in each cancer subtype. The results showed that the smaller the network-based proximity, the more the overlapping number of communities and the closer the biological and pharmacological relationships of the two drugs (Figures 1C, D). Also, the results showed that the bigger the network-based proximity, the less the overlapping number of communities and the more distant the biological and pharmacological relationships of the two drugs. For example, Bortezomib is the first proteasome inhibitor approved by US FDA to treat multiple myeloma. Dihydroartemisinin was reported to induce autophagy and apoptosis of multiple myeloma cell lines (Wu et al., 2020).

**FIGURE 2:**
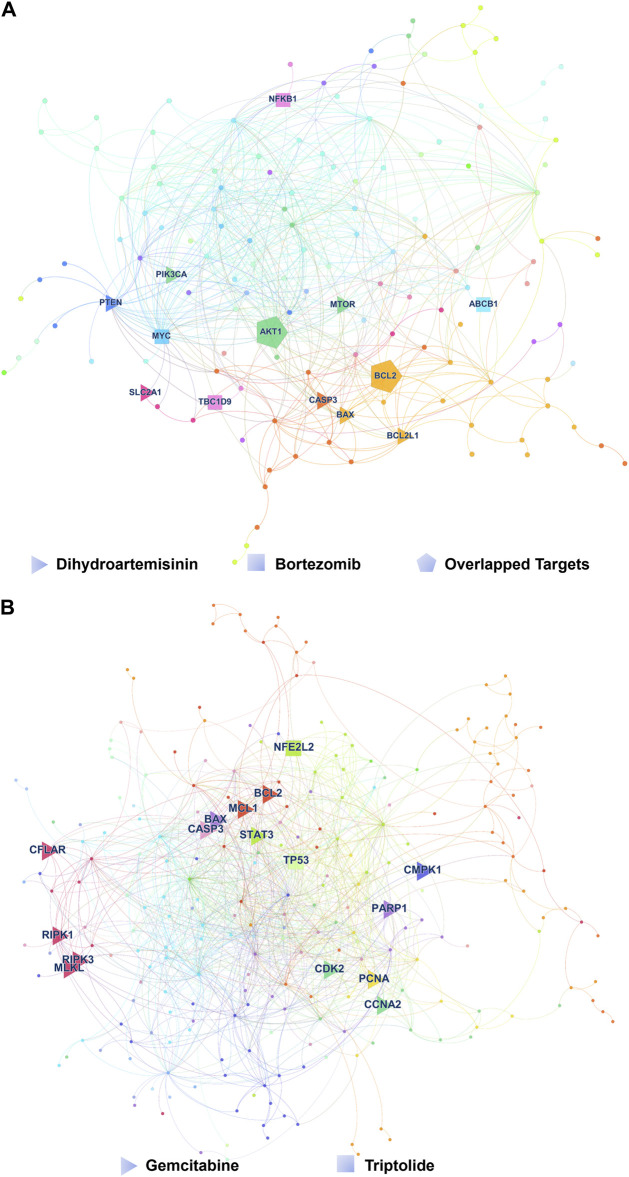
Typical drug combinations in AML and NSCLS. **(A)** The target proteins hit by Bortezomib and Dihydroartemisinin in the gene network of AML. The color of each node represents the network community to which the corresponding gene of the target protein belongs. Triangles represent the target proteins of Dihydroartemisinin, squares represent the target proteins of Bortezomib Associations between molecular function similarity of target proteins from Gene Ontology annotations and network-based proximity in 12 cancer types, and pentagons represent the common target proteins of Bortezomib and Dihydroartemisinin. **(B)** The target proteins hit by Gemcitabine and Triptolide in the gene network of NSCLC. Triangles represent the target proteins of Gemcitabine, and squares represent the target proteins of Triptolide.

As shown in Figure 2A, Bortezomib’s targets were in the same network neighborhood as the targets of Dihydroartemisinin in the AML network, and the network-based proximity between the targets was −0.334. Community detection further showed Bortezomib and Dihydroartemisinin shared one community, suggesting their biological and functional similarities. On the other hand, Gemcitabine is a nucleoside analogue with activity in NSCLC. Triptolide is a promising agent for NSCLC, which suppresses cancer growth and metastasis by inhibiting β-catenin-mediated epithelial-mesenchymal transition. In the NSCLC network, the targets of Gemcitabine were in a topologically distinct neighborhood from the targets of Triptolide (Figure 2B), having a network-based proximity of 0.205. No overlapping communities were found between the targets of the two drugs. The results indicated lower similarities in the biological and functional profiles between Gemcitabine and Triptolide.

Based on the overlapping communities (≥1) and the network-based proximity (<0), we identified 61,754 combinations for NSCLC, 65,838 for colon cancer, 50,457 for AML, 63,226 for pancreatic cancer, 102,771 for breast cancer, 64,114 for ovarian cancer, 71,464 for liver cancer, 60,463 for prostate cancer, 45,432 for osteosarcoma, 41,459 for glioma and 67,627 for hepatocellular cancer (Supplementary Tables S1–S11). We found that 19,031 combinations appeared in more than 10 cancers, 39,079 in more than six cancers, and 70,015 uniquely synergistic combinations. This indicated the similar and specific characteristics of different cancers. For example, Bortezomib was the first proteasome inhibitor to be approved by the US FDA. Topotecan was a chemotherapy drug. Bortezomib and Topotecan was predicted to be combinational in 11 cancer types. This indicated the similarity of different cancer types. In addition, Bruceine D is a major bioactive component isolated from the traditional Chinese medicinal plant Brucea javanica. Harmine is a β-carboline alkaloid found in multiple medicinal plants. The combinational effect of Bruceine D and Harmine was only found in NSCLC.

### 3.2 Network configurations of 11 cancer types

To further understand the difference and similarity of cancer types, we analyzed the biological signaling pathways in cancer types. Betweenness centrality was used to describe the importance of nodes in a network in terms of the fraction of shortest paths that pass through them. For each cancer subtype network, betweenness centrality for each node was calculated and sequenced. In this study, nodes with higher betweenness centrality (nodes at top 60%) were identified as important genes for each cancer subtype network. Within the important genes, gene frequency was further calculated, and genes co-occurred in more than ten cancer types were identified as hot cancer genes, which included *MYC, AKT1, BCL2, MCL1, CASP8, BAX, MAPK8, CDKN1A, PCNA, MTOR, PTEN, BCL2L11, VEGFA, FASN, PARP1, PIK3R1, EGFR, CDK2, JUN, CASP3, BCL2L1, FLT4, XIAP, BAD, PRKAA1, PRKAB1, CDK1, STAT3, BECN1, and MAP1LC3B*. As shown in Table 1, these genes were enriched in pathways promoting carcinogenesis, proliferation, invasion, and metastasis of tumor cells and pathways inhibiting cancer proliferation and growth. It was reported that acting on multiple targets inside the cancer cell with several biological mechanisms was favorable to overcome drug resistance (Karges et al., 2020). Supplementary Table S12 showed drugs targeted to more than two hot cancer genes in each cancer network. We found many targeted drugs such as Metformin, Celecoxib, and Sorafenib repeatedly appeared in difference cancers. This implied that these drugs probably had higher potency to overcome drug resistance, compared with other drugs that acted on less targets.

**TABLE 1:** Important biological signaling pathways occurring in more than 10 cancer types.

Promoting carcinogenesis, proliferation, invasion, and metastasis of tumor cells		
Pathway	FDR	Genes
PI3K-Akt signaling pathway	2.9E-16	CDKN1A, HSP90AA1, PRKAA1, CDKN1B, BAD, FLT4, PTEN, PIK3R1, EGFR, MTOR, PTK2, VEGFA, BCL2L11, CCND1, CDK4, MYC, CDK2, BCL2, AKT1, MCL1, BCL2L1
JAK-STAT signaling pathway	6.74E-09	CDKN1A, CCND1, MYC, STAT3, BCL2, AKT1, PIK3R1, EGFR, MTOR, BCL2L1, MCL1
HIF-1 signaling pathway	5.61E-08	CDKN1A, CDKN1B, STAT3, BCL2, AKT1, PIK3R1, EGFR, MTOR, VEGFA
Insulin signaling pathway	4.34E-06	PRKAA1, MAPK8, BAD, FASN, AKT1, PIK3R1, PRKAB1, MTOR
VEGF signaling pathway	9.94E-06	SRC, BAD, AKT1, PIK3R1, PTK2, VEGFA
TNF signaling pathway	1.72E-05	JUN, MAPK8, CASP8, CASP3, AKT1, PIK3R1, BIRC3
Adipocytokine signaling pathway	2.15E-05	PRKAA1, MAPK8, STAT3, AKT1, PRKAB1, MTOR
Prolactin signaling pathway	2.31E-05	MAPK8, CCND1, SRC, STAT3, AKT1, PIK3R1
Neurotrophin signaling pathway	2.44E-05	JUN, MAPK8, BAD, BCL2, BAX, AKT1, PIK3R1
Thyroid hormone signaling pathway	2.68E-05	CCND1, SRC, BAD, MYC, AKT1, PIK3R1, MTOR
Relaxin signaling pathway	3.85E-05	JUN, MAPK8, SRC, AKT1, PIK3R1, EGFR, VEGFA
Ras signaling pathway	0.000145	MAPK8, BAD, FLT4, AKT1, PIK3R1, EGFR, BCL2L1, VEGFA
MAPK signaling pathway	0.000557	JUN, MAPK8, MYC, CASP3, FLT4, AKT1, EGFR, VEGFA
IL-17 signaling pathway	0.001183	HSP90AA1, JUN, MAPK8, CASP8, CASP3
NF-kappa B signaling pathway	0.001721	PARP1, BCL2, XIAP, BCL2L1, BIRC3
mTOR signaling pathway	0.007371	PRKAA1, PTEN, AKT1, PIK3R1, MTOR
Wnt signaling pathway	0.052484	JUN, MAPK8, CCND1, MYC
B cell receptor signaling pathway	0.062964	JUN, AKT1, PIK3R1
Inhibiting cancer proliferation and growth		
FoxO signaling pathway	2.12E-12	CDKN1A, PRKAA1, CDKN1B, STAT3, PTEN, PIK3R1, PRKAB1, EGFR, MAPK8, BCL2L11, CCND1, CDK2, AKT1
AMPK signaling pathway	2.68E-05	PRKAA1, CCND1, FASN, AKT1, PIK3R1, PRKAB1, MTOR
GnRH signaling pathway	0.011011	JUN, MAPK8, SRC, EGFR

Further, we found unique pathways in different cancer types by enriching gene sets that co-occurred in less than four cancer types, i.e., RIG-I-like receptor signaling pathway for colorectal cancer (FDR = 0.02), Notch signaling pathway for AML (FDR = 0.08), TGF-beta signaling pathway for pancreatic cancer (FDR = 0.04), Apelin signaling pathway for liver cancer (FDR = 0.01), prolactin signaling pathway (FDR = 0.03) for prostate cancer. For RIG-I-like receptor signaling pathway in colorectal cancer, MAPK9, MAPK14, MAP3K7, and RELA were enriched in the pathway. The results indicated the specificity of different cancer types.

### 3.3 Validation of network-based drug combination design strategy

We selected four types of cancer - colorectal cancer, breast cancer, pancreatic cancer, and AML - to assess the effectiveness of our prediction model. To evaluate the impact of combination treatment on apoptosis, we conducted Annexin V/PI based flow cytometry analysis after administering drug treatments to the chosen cancer cells.

**FIGURE 3:**
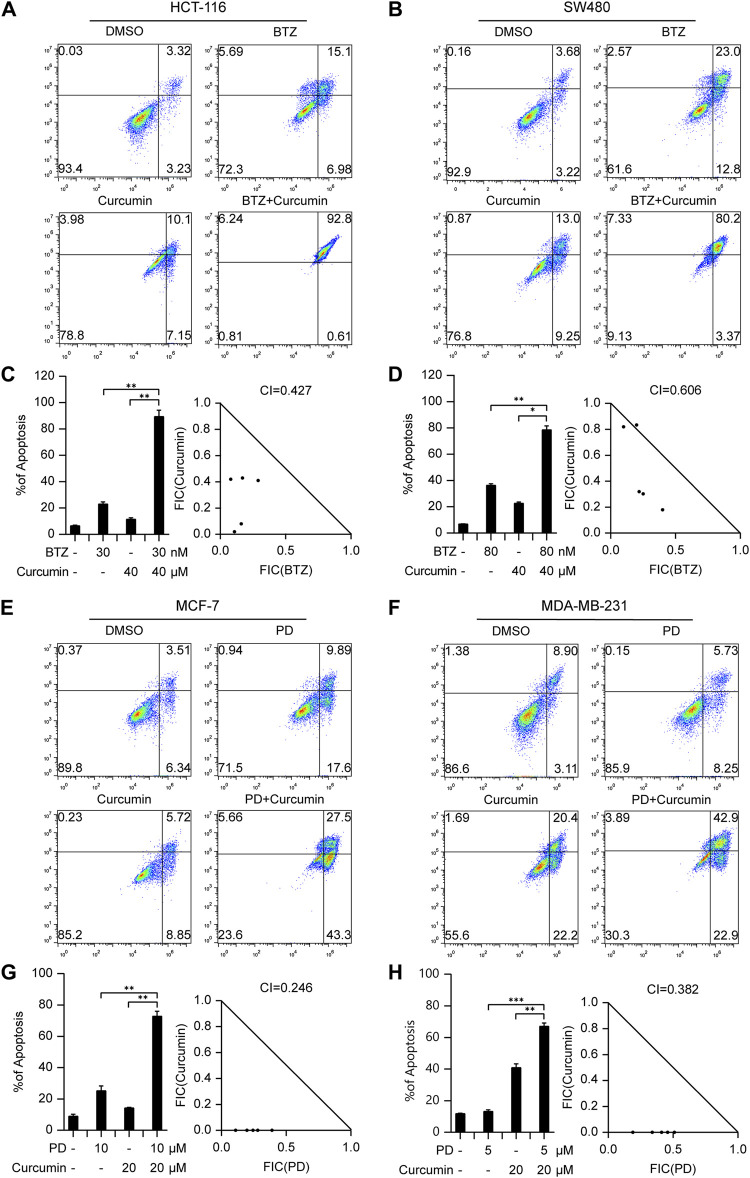
The drug combinations selected from our predication model induce synergistic lethality in colorectal cancer and Breast cancer cells. Annexin V flow cytometry of HCT116 **(A)** or SW480 **(B)** cells underwent treatment of Bortezomib (30 nM for HCT116, 80 nM for SW480), Curcumin (40 μM), alone or in combination for 48 h **(C,D)** Apoptotic cell death was analyzed and shown on the left. Combination Index (CI) of Bortezomib and Curcumin was analyzed in colorectal cancer cells using the Compusyn software. Annexin V flow cytometry of MCF-7 **(E)** or MDA-MB-231 **(F)** cells were subjected to administration of 10 or 5 μM Palbociclib, 20 μM Curcumin, alone or in combination for 48 h **(G,H)** Apoptotic cell death was analyzed and shown on the left. CI of Bortezomib and Curcumin was analyzed in breast cancer cells using the Compusyn software on the right. Data shown represent the means (±SEM) of biological triplicates. *p < 0.05, **p < 0.01, ***p < 0.001.

For colorectal cancer, we investigated the combined effects of Bortezomib and Curcumin. This combination had not been reported previously. Bortezomib is an FDA-approved proteasome inhibitor used in the first-line treatment of multiple myeloma. Curcumin, derived from the herb Curcuma longa, exhibits anti-cancer properties against colorectal cancer through various cellular mechanisms (Weng and Goel, 2022). In our study, we treated HCT116 and SW480 cells with Bortezomib, Curcumin, or a combination of both. As anticipated, the Bortezomib/Curcumin combination synergistically enhanced the sensitivity of colorectal cancer cells to Curcumin-induced cell death at a concentration that did not cause significant toxicity when administered individually (Figures 3A, B; Supplementary Figures S12A, B). To further validate the synergistic effects, we utilized CompuSyn software, which employs the Chou-Talalay method to calculate the combination index (CI). This method provides a quantitative measure of synergistic (CI < 1), additive (CI = 1), and antagonistic (CI > 1) effects (Chou, 2010). As depicted in Figures 3C, D, the CI values for HCT116 and SW480 cells were 0.427 and 0.606, respectively, further confirming the synergism between Bortezomib and Curcumin.

In breast cancer, we examined the combination of Palbociclib (CDK4/6 inhibitor) and Curcumin, a novel approach that has not been previously explored in other types of cancer. Our results demonstrated a strong synergistic effect between Palbociclib and Curcumin in estrogen receptor (ER) positive breast cancer cell line MCF-7 as well as triple-negative breast cancer cell line (TNBC) MDA-MB-231. The corresponding CI values for MCF-7 and MDA-MB-231 cells were 0.246 and 0.382, respectively, indicating a promising therapeutic option for breast cancer treatment (Figures 3E–H; Supplementary Figures S12C, D).

**FIGURE 4:**
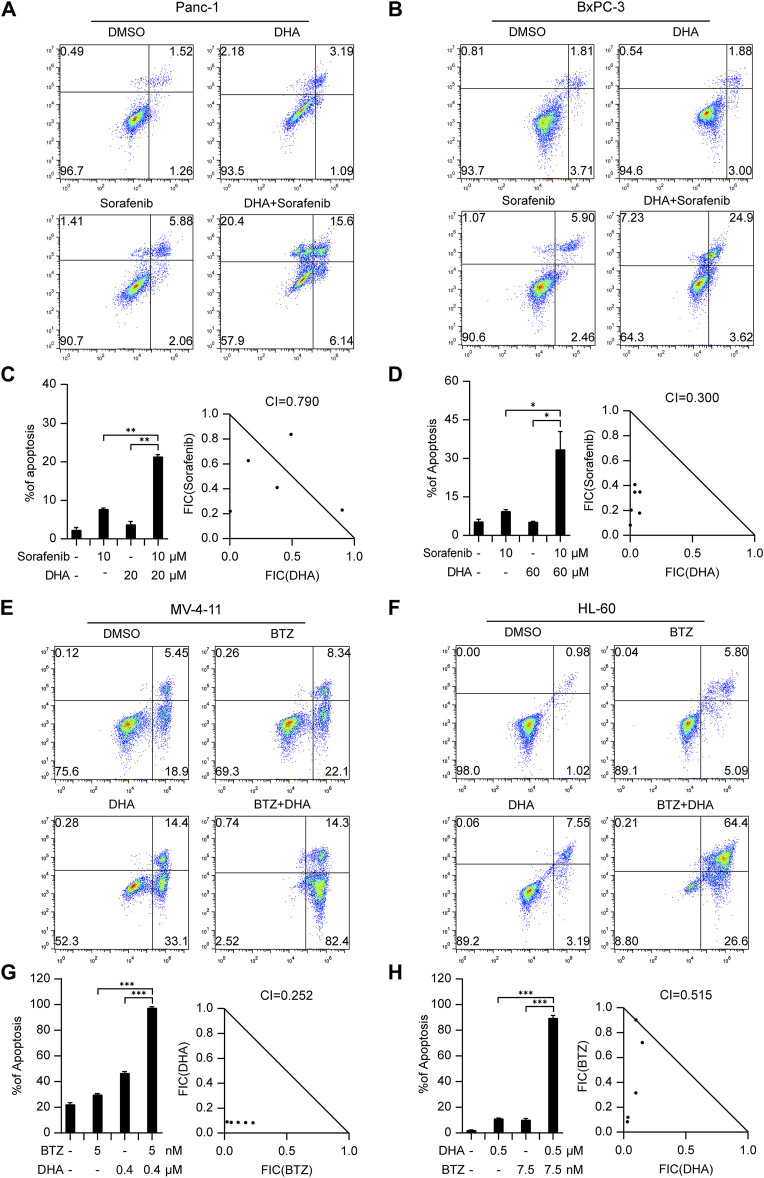
The drug combinations selected from our predication model induce synergistic lethality in pancreatic cancer and AML cells. Annexin V flow cytometry of Panc-1 **(A)** and BxPC-3 **(B)** underwent treatment of Dihydroartemisinin (20 μM for Panc-1, 60 μM for BxPC-3), Sorafenib (10 μM), alone or in combination for 48 h **(C,D)** Apoptotic cell death was analyzed and shown on the left. CI of Dihydroartemisinin and Sorafenib was analyzed in pancreatic cancer cells using the Compusyn software on the right. Annexin V flow cytometry of MV4-11 **(E)** and HL-60 **(F)** were subjected to administration of five or 7.5 nM Bortezomib, 400 or 500 nM Dihydroartemisinin, alone or in combination for 48 h **(G,H)** Apoptotic cell death was analyzed and shown on the left. CI of Bortezomib and Dihydroartemisinin was analyzed in AML cells. Data shown represent the means (±SEM) of biological triplicates. *p < 0.05, **p < 0.01, ***p < 0.001.

Pancreatic cancer poses considerable difficulties due to its high mortality rate and limited treatment options. In an effort to tackle this issue, we explored the potential of combining Dihydroartemisinin, a natural herbal product, with the tyrosine kinase inhibitor sorafenib. Our findings revealed a strong synergistic effect of this combination in inducing cell death in Panc-1 and BxPC-3 cells (Figures 4A, B; Supplementary Figures S12E, F). Notably, the respective CI values for Panc-1 and BxPC-3 cells were 0.790 and 0.3 (Figures 4C, D), further highlighting the promising therapeutic potential of this approach.

In the context of hematological malignancies, we focus was on AML, which is the most prevalent malignant myeloid disorder in adults. An innovative combination therapy involving Dihydroartemisinin and Bortezomib, not previously explored in leukemia, was investigated. The synergistic effects of this combination were evaluated in MV4-11 and HL-60 cells. After determining effective concentrations (Supplementary Figures S12G, H), a fixed concentration of Dihydroartemisinin and Bortezomib was administered respectively to induce apoptosis. As illustrated in Figures 4E, F, the combination of Dihydroartemisinin and Bortezomib significantly enhanced apoptosis compared to either drug treatment alone. The respective CI values for MV4-11 and HL-60 cells were 0.252 and 0.515 (Figures 4G, H). Overall, our results demonstrate that the selected combination therapy groups, determined using our prediction model, exhibited synergistic effects in different types of cancer cells, including solid tumors and hematological malignancies.

A critical aspect of our selection process was to identify drug combinations that have not been previously reported for the treatment of the specified types of tumors, thereby ensuring the novelty and potential impact of our findings. From the extensive list of 1,317 drugs and the subsequent tens of thousands of combinations predicted by our network-based strategy, we handpicked a diverse subset for experimental validation. This subset was chosen based on unique network proximity measures and community overlap, indicative of potential synergistic interactions that have not been explored in the context of the tumor types we studied.

Our drug selection process was grounded in scientific literature, bioinformatics predictions, and the interdisciplinary expertise of our team. We aimed to explore a broad spectrum of potential drug combinations, leading us to a systematic and unbiased selection of drugs that not only included established anticancer treatments but also encompassed compounds traditionally used for other diseases and natural agents with potential anti-cancer activity.

Furthermore, we used the confusion matrix method to measure the performance of our prediction strategy, i.e., when S_AB_ < 0 and community overlap ≥1 for two drugs, we defined the two drugs were combinational. The accuracy, sensitivity and specificity were 78.9%, 75.0% and 80.0%, showing good agreement between the predicted and the experimental combination drugs (Table 2). The result validated the reliability of our drug combination design strategy. As shown in Supplementary Table S13, we further used five statistics methods to estimate the 95% confidence interval of accuracy, sensitivity and specificity. The lower bounds of the 95% prediction interval of accuracy were always more than 0.544, which indicated that the drug interaction prediction strategy proposed in this study only has a probability of less than 5% of false positive conclusions due to insufficient experimental results. The upper bounds of the 95% prediction interval of accuracy were always more than 0.915, which indicated the great application potential of our drug interaction prediction strategy. Similar results were found for the 95% confidence interval of specificity, i.e., the upper and the lower bounds of the 95% confidence interval of specificity were more than 0.986 and 0.640, respectively. Reliable specificity helped to distinguish negative results from a large number of combination hypotheses, which was crucial for reducing false positive results in high-throughput testing and saving drug combination development costs.

**TABLE 2:** Comparison of predicted and experimental combination drugs.

Drug A	Drug B	S_AB_	Community overlap	Prediction	Ground truth
Pancreatic cancer					
Sorafenib	Dihydroartemisinin	−0.142	1	True	True
Non-small cell lung cancer					
Triptolide	Curcumin	0.230	0	False	False
Gemcitabine	Triptolide	0.205	0	False	False
Gemcitabine	Tanshinone IIA	0.716	0	False	False
Osimertinib	Tanshinone IIA	0.743	0	False	False
Metformin	Gemcitabine	−0.077	0	False	False
Metformin	Tanshinone IIA	0.713	1	False	False
Gemcitabine	Osimertinib	0.042	1	False	False
Dihydroartemisinin	Metformin	0.063	0	False	False
Breast cancer					
Bortezomib	Curcumin	−0.071	0	False	False
Curcumin	Gemcitabine	−0.202	1	True	False
Bortezomib	Dihydroartemisinin	−0.334	1	True	False
Gemcitabine	Palbociclib	0.281	1	False	False
Palbociclib	Curcumin	0.287	0	False	True
Curcumin	Sorafenib	−0.376	1	True	False
Colorectal Cancer					
Curcumin	Bortezomib	−0.071	1	True	True
Acute myeloid leukemia					
Bortezomib	Dihydroartemisinin	−0.334	2	True	True
Decitabine	Palbociclib	0.598	0	False	False
Bortezomib	Palbociclib	0.164	1	False	False

To evaluate the robustness of network-based prediction models, we tested different α , β , and γ (Supplementary Table S14). The results showed that the accuracy of network-based prediction models changed between 63.1% and 84.2%. When α and β were set to 10 and 0.2, the accuracy of prediction was 84.2%, sensitivity 50.0%, and specificity 93.3%. When α and β were set to 10 and 0.15, sensitivity and specificity achieved best balance, and thus α=10 and β=0.15 were chose to predict drug combination.

## 4 Discussion

The proximity measure of the target network used in this study is an algorithm based on the network topology, which simultaneously considers the dispersion within the two target sets of the two drugs and the shortest distance between the target sets. In the PPI network, edges represent direct interactions between targets, so the proximity measure indicates the average path length required for drug targets to interact. Obviously, the smaller the proximity measure, the easier it is for drug combinations to produce synergistic effects. On the other hand, whether two drugs can produce a combined effect in the treatment of a specific disease also depends on the function of the targets. If the drug targets are not disease-related and no shortest path can be found between the targets of the two drugs, it is difficult for the two drugs to have a joint effect on the treatment of a specific disease. Therefore, we use the cancer stratified target network and its community division to ensure the disease specificity of drug combination effect. The community size threshold (|C_i|≥3) helped to reduce the occurrence of false positive results, which provided promising predictive ability for drug combination. The result was validated by our cellular experiments involving multiple cancers.

In addition, we used our prediction strategy to identify 61,754 combinations for NSCLC. To validate the prediction, we randomly selected four paris of drugs. However, we did not observe any drug pairs that showed a synergistic effect on NSCLC in the cellular experiment. It was worth noticing that Dihydroartemisinin was the only drug that hit multiple hot cancer genes on the NSCLC cancer stratification network, showing its potential to non-specifically interact with other NSCLC chemotherapeutic drugs. We believe that Dihydroartemisinin is worth further research to verify if it can produce drug synergy effects with other specific anticancer drugs, to improve the prognosis of NSCLC patients or relieve their drug resistance.

In future studies, we intend to explore a more accurate and efficient strategy for drug combination prediction. To determine the specific effects of drug combinations, such as synergistic, antagonistic, or toxic effects, we plan to model the direction of drug-target interactions by building directed graphs on existing networks, incorporating drug-target isoform networks, or introducing new node attributes. Furthermore, we will incorporate the validated hypothesis from this study into more advanced algorithms, including various deep learning techniques, to enhance predictive ability for practical applications. Additionally, the availability of reliable experimental or clinically derived drug interaction datasets remains limited. Therefore, constructing a comprehensive drug interaction database is necessary to facilitate benchmarking between different algorithms and advance research in drug combination discovery.

There are still some limitations to this study. Although we made efforts to obtain a comprehensive literature set and used text mining techniques for information extraction, there may still be missed information due to the limitations of text mining algorithms, leading to false negatives. To ensure result reliability, we supplemented the drug-target relationships with data from Drugbank. While our prediction method demonstrates high specificity and low probability of producing false negatives, there is still a wide confidence interval for the performance indicators. We conducted experiments on multiple cancers and drug combinations to validate the predicted results; however, further validation on a larger scale or a well-designed benchmark dataset is necessary for practical application of this method, as mentioned earlier.

In conclusion, this study developed a drug combination prediction strategy based on complex biological networks and demonstrated its effectiveness through experimental research. The results also provide insights for future directions in anticancer drug combination research. This prediction strategy holds potential for widespread application in discovering drug combinations for cancer and other diseases to improve patient prognosis, overcome drug resistance, and mitigate adverse drug reactions.

## Data Availability

The raw data supporting the conclusions of this article will be made available by the authors, without undue reservation.
