# Corrigendum: Network-based prediction of anti-cancer drug combinations

**DOI:** 10.3389/fphar.2024.1495634

**Published:** 2024-11-04

**Authors:** Jue Jiang, Xuxu Wei, YuKang Lu, Simin Li, Xue Xu

**Affiliations:** ^1^ School of Medicine, Wuhan University of Science and Technology, Wuhan, Hubei, China; ^2^ Key Laboratory of Chinese Internal Medicine of Ministry of Education, Dongzhimen Hospital, Beijing University of Chinese Medicine, Beijing, China

**Keywords:** cancer, drug combination, protein-protein interaction network, network proximity, community detection

In the published article, there was an error. In the **Formula** used to define the target community of drug X, some symbols were not properly displayed. Additionally, the description of the parameter α was missing.

A correction has been made to **2 Materials and methods**, *2.4 Drug combination prediction in cancer-specific network*, Paragraph 2. This sentence previously stated:

$$“C_{X}=\left\{C_{j}\left|C_{j}\cap T_{X}\right|\geq \min\left(\alpha ,\beta ,\left|C_{j}\right|\right),\left|C_{j}\right|\geq \gamma ,\;C_{j}\in C\right\}$$

Where $C_{X}$ represents the community hit by the drug X, $C_{j}$ is the community within $G_{ca}$ , $T_{X}$ is the target set hit by the drug X. β is a constant between 0 and 1, representing what proportion of drug targets in a community can be considered to hit the community.”

The corrected sentence appears below:

$$“C_{X}=\left\{C_{j}|\left|C_{j}\cap T_{X}\right|\geq \min\left(\alpha ,\beta \left|C_{j}\right|\right),\left|C_{j}\right|\geq \gamma ,\;C_{j}\in C\right\}$$

Where $C_{X}$ represents the community hit by the drug X, $C_{j}$ is the community within $G_{ca}$ , $T_{X}$ is the target set hit by the drug X. α (greater than 0) represents the number of targets within the community that the drug must hit, while β (between 0 and 1) representing the proportion of drug targets that must be hit for the community to be considered as hit.”

The authors apologize for this error and state that this does not change the scientific conclusions of the article in any way. The original article has been updated.

