# Supplementary material for: Network-based prediction of anti-cancer drug combinations
Source: Front Pharmacol. 2024 Aug 28;15:1418902. doi: 10.3389/fphar.2024.1418902 (PMC11357946; doi:10.3389/fphar.2024.1418902)
Supplement: Supplementary file 5 [file Table12.DOCX]

**supplementary Table 12. drugs hitting on hot cancer genes (>2) in cancer subnetworks**

| Drug | Hot cancer gene hits | Hits | Cancer co-occurrence |
| --- | --- | --- | --- |
| Acute myeloid leukemia | | |  |
| metformin | CDKN1A, PRKAA1, PRKAB1 | 3 | 6 |
| bortezomib | BCL2, CASP8, FASN, CASP3 | 4 | 2 |
| Breast Cancer | | |  |
| metformin | AKT1, BCL2, BAX, CDKN1A, PCNA, PTEN, BAD, STAT3 | 8 | 6 |
| paclitaxel | AKT1, BCL2, BAX, PARP1, CASP3 | 5 | 2 |
| Colorectal Cancer | | |  |
| metformin | BCL2, FASN, PRKAA1, PRKAB1 | 4 | 6 |
| celecoxib | CDKN1A, PCNA, CASP3 | 3 | 4 |
| gossypol | BCL2, MCL1, BAX, PCNA, MTOR, PARP1, CASP3, BCL2L1 | 8 | 2 |
| azd8055 | BCL2, MCL1, BCL2L11, BCL2L1 | 4 | 2 |
| Glioma | | |  |
| celecoxib | BCL2, BAX, PARP1, CASP3 | 4 | 4 |
| Hepatocellular cancer | | |  |
| metformin | AKT1, BCL2, BAX, MTOR, PTEN, CASP3, PRKAA1, PRKAB1 | 8 | 6 |
| sorafenib | AKT1, CASP8, MTOR, CASP3, PRKAA1, PRKAB1, STAT3 | 7 | 4 |
| celecoxib | AKT1, MTOR, PRKAA1, PRKAB1 | 4 | 4 |
| tanshinone iia | BCL2, BAX, CASP3 | 3 | 2 |
| idelalisib | BCL2, BCL2L11, BCL2L1 | 3 | 2 |
| telmisartan | MTOR, PRKAA1, PRKAB1 | 3 | 2 |
| Liver cancer | | |  |
| metformin | AKT1, BCL2, BAX, MTOR, PTEN, CASP3, PRKAA1, PRKAB1 | 8 | 6 |
| sorafenib | AKT1, MCL1, CASP8, PCNA, MTOR, CASP3, PRKAA1, PRKAB1, STAT3 | 9 | 4 |
| celecoxib | AKT1, MTOR, PRKAA1, PRKAB1 | 4 | 4 |
| tanshinone iia | BCL2, BAX, CASP3 | 3 | 2 |
| idelalisib | BCL2, BCL2L11, BCL2L1 | 3 | 2 |
| telmisartan | MTOR, PRKAA1, PRKAB1 | 3 | 2 |
| Non-small cell lung cancer | | |  |
| dihydroartemisinin | BCL2, MCL1, MTOR, BCL2L11, BCL2L1, STAT3 | 6 | 3 |
| Osteosarcoma | | |  |
| sorafenib | MTOR, PRKAA1, PRKAB1 | 3 | 4 |
| bortezomib | MYC, BCL2, PCNA, STAT3 | 4 | 2 |
| Ovarian cancer | | |  |
| metformin | BCL2, BAX, XIAP | 3 | 6 |
| dihydroartemisinin | BCL2, BAX, BAD | 3 | 3 |
| cisplatin | AKT1, BCL2, BAX, MTOR, PARP1, CASP3, BECN1, MAP1LC3B | 8 | 2 |
| azd8055 | BCL2, MCL1, MTOR, BCL2L11, BCL2L1 | 5 | 2 |
| paclitaxel | AKT1, BCL2, PCNA, STAT3 | 4 | 2 |
| Pancreatic Cancer | | |  |
| dihydroartemisinin | BCL2, BAX, CDKN1A, PCNA, CDK2 | 5 | 3 |
| Prostate cancer | | |  |
| sorafenib | AKT1, MCL1, PCNA, CDK2 | 4 | 4 |
| gossypol | BCL2, CASP8, PCNA, CASP3 | 4 | 2 |
| cisplatin | AKT1, CASP8, CASP3, XIAP | 4 | 2 |
