# Supplementary material for: Network-based prediction of anti-cancer drug combinations
Source: Front Pharmacol. 2024 Aug 28;15:1418902. doi: 10.3389/fphar.2024.1418902 (PMC11357946; doi:10.3389/fphar.2024.1418902)
Supplement: Supplementary file 9 [file Table13.docx]

**Supplementary Table 13. Estimation of Predictive Performance Using**

**Different Statistical Methods**

| Statistic method | Accuracy (95% CI) | Specificity (95% CI) | Sensitivity (95% CI) |
| --- | --- | --- | --- |
| Exact^a^ | 0.789 (0.544 to 0.939) | 0.800 (0.640 to 0.998) | 0.750 (0.118 to 0.882) |
| Wilson^b^ | 0.789 (0.567 to 0.915) | 0.800 (0.667 to 0.986) | 0.750 (0.188 to 0.812) |
| Agresti^c^ | 0.789 (0.561 to 0.920) | 0.800 (0.646 to 1.007) | 0.750 (0.188 to 0.812) |
| Clopper-Pearsons^d^ | 0.789 (0.544 to 0.939) | 0.800 (0.640 to 0.998) | 0.750 (0.118 to 0.882) |
| Jeffreys^e^ | 0.789 (0.574 to 0.924) | 0.800 (0.693 to 0.992) | 0.750 (0.167 to 0.833) |

a: Collett D (1999). Modelling Binary Data. Chapman & Hall/CRC, Boca Raton Florida, pp. 24.

b: Epidemiology An Introduction. Oxford University Press, London, pp. 164 - 175.

c: Agresti A, Coull B (1998). Approximate is better than 'exact' for interval estimation of binomial proportions. The American Statistician 52. DOI: 10.2307/2685469.

d: Clopper C, Pearson E (1934) The use of confidence or fiducial limits illustrated in the case of the binomial. Biometrika 26: 404 - 413. DOI: 10.1093/biomet/26.4.404.

e: Brown L, Cai T, Dasgupta A (2001). Interval estimation for a binomial proportion. Statistical Science 16: 101 - 133.
