# Supplementary material for: Network-based prediction of anti-cancer drug combinations
Source: Front Pharmacol. 2024 Aug 28;15:1418902. doi: 10.3389/fphar.2024.1418902 (PMC11357946; doi:10.3389/fphar.2024.1418902)
Supplement: Supplementary file 15 [file DataSheet1.DOCX]

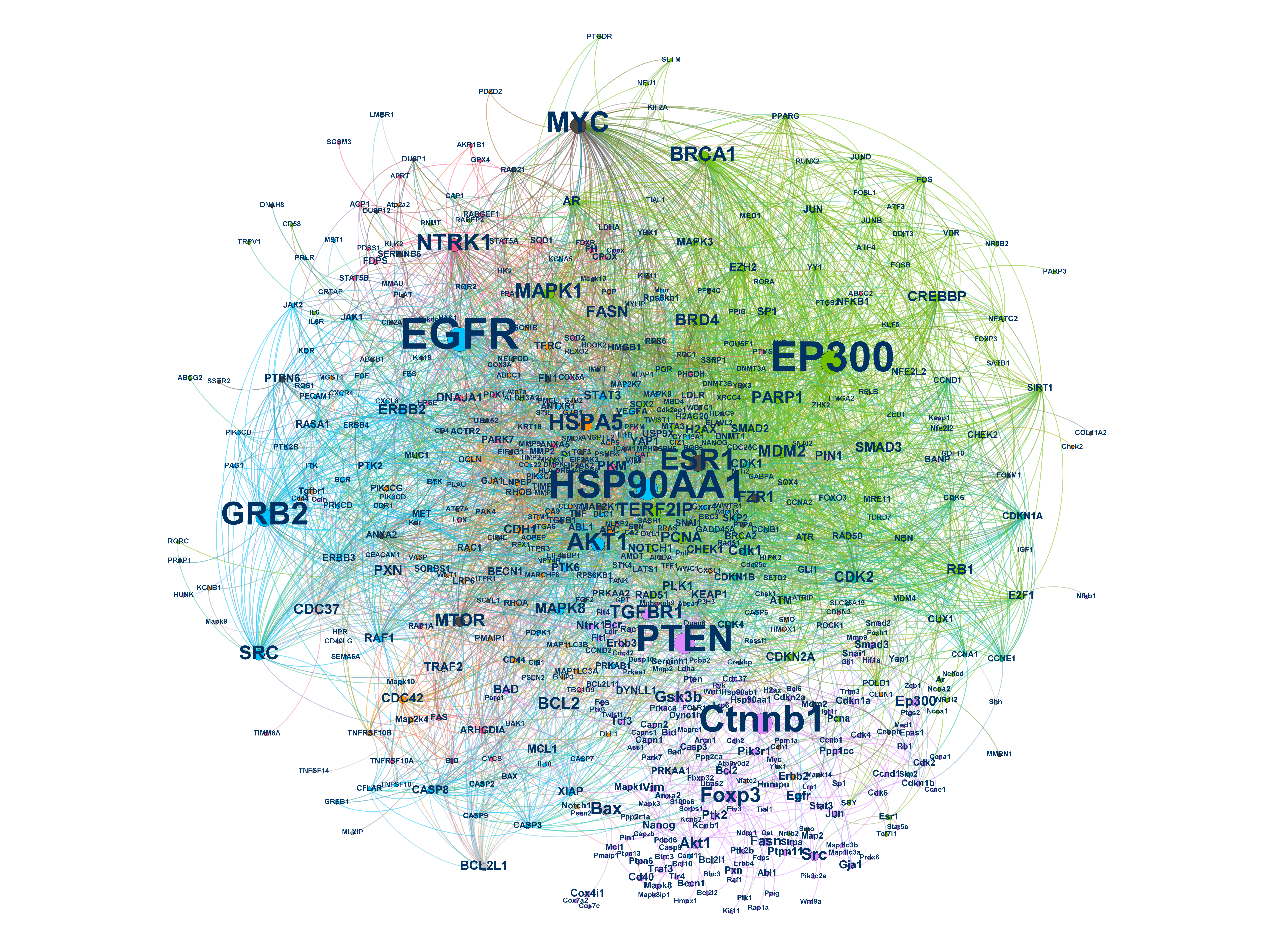


**Figure S1 Colon cancer network. Each node represents a gene.** The size of node indicates the importance of the gene in the network. The color of the nodes represents the network communities to which the genes belong. The edges between two nodes represent potential interactions between genes.


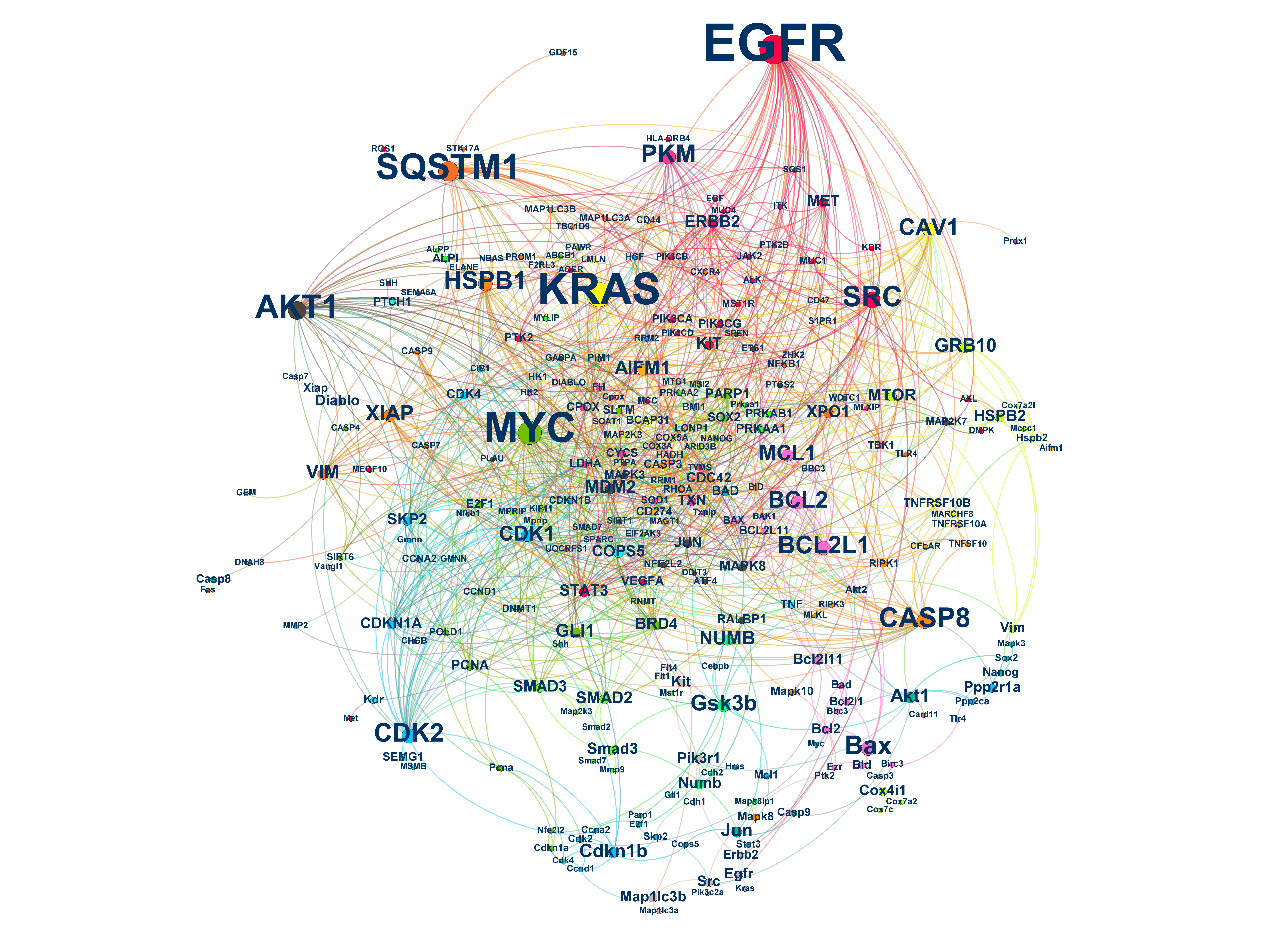


**Figure S2** **Pancreatic cancer network.** Each node represents a gene. The size of node indicates the importance of the gene in the network. The color of the nodes represents the network communities to which the genes belong. The edges between two nodes represent potential interactions between genes.


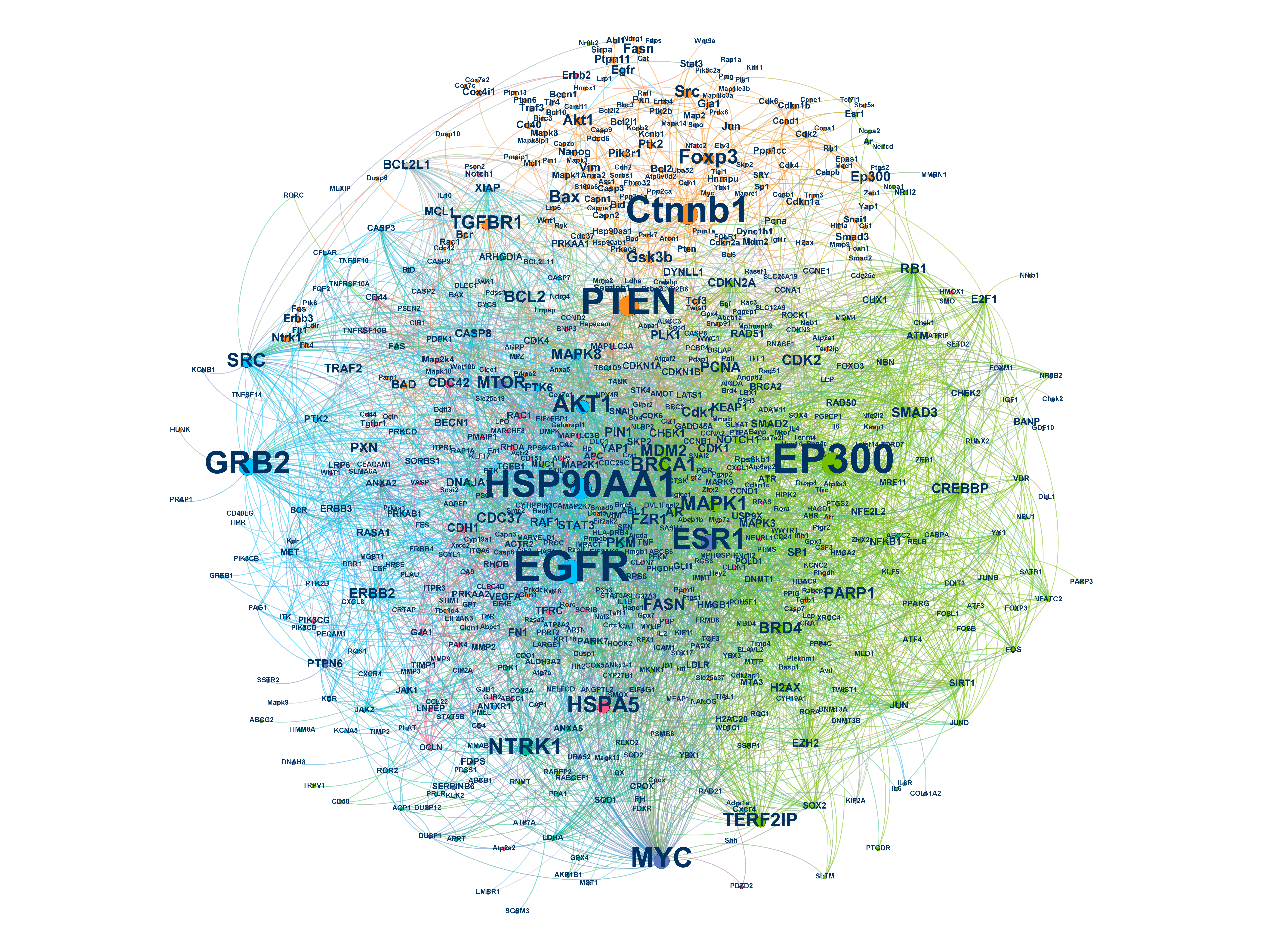


**Figure S3 Breast cancer network.** Each node represents a gene. The size of node indicates the importance of the gene in the network. The color of the nodes represents the network communities to which the genes belong. The edges between two nodes represent potential interactions between genes.


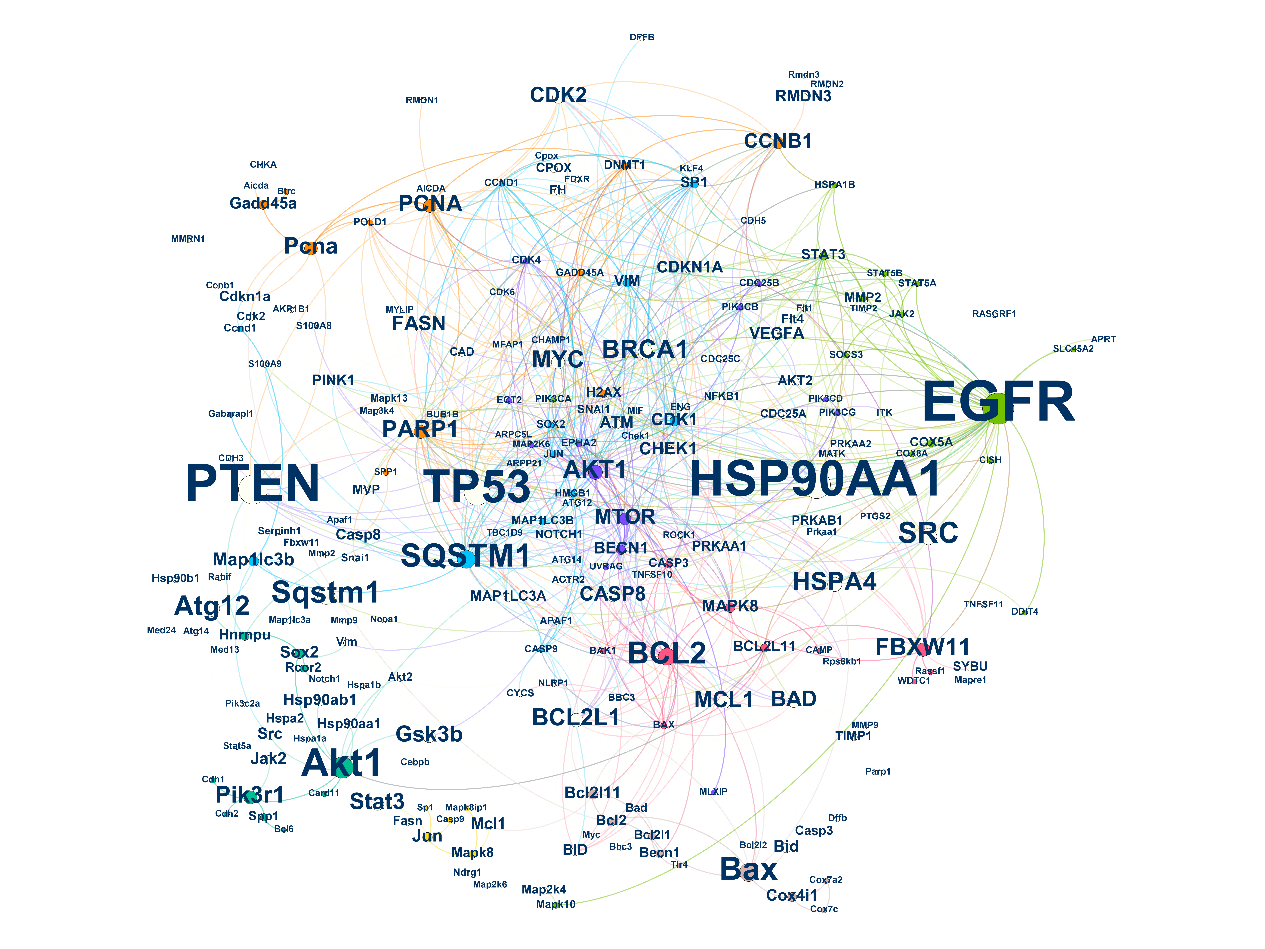


**Figure S4 Ovarian cancer network.** Each node represents a gene. The size of node indicates the importance of the gene in the network. The color of the nodes represents the network communities to which the genes belong. The edges between two nodes represent potential interactions between genes.

**
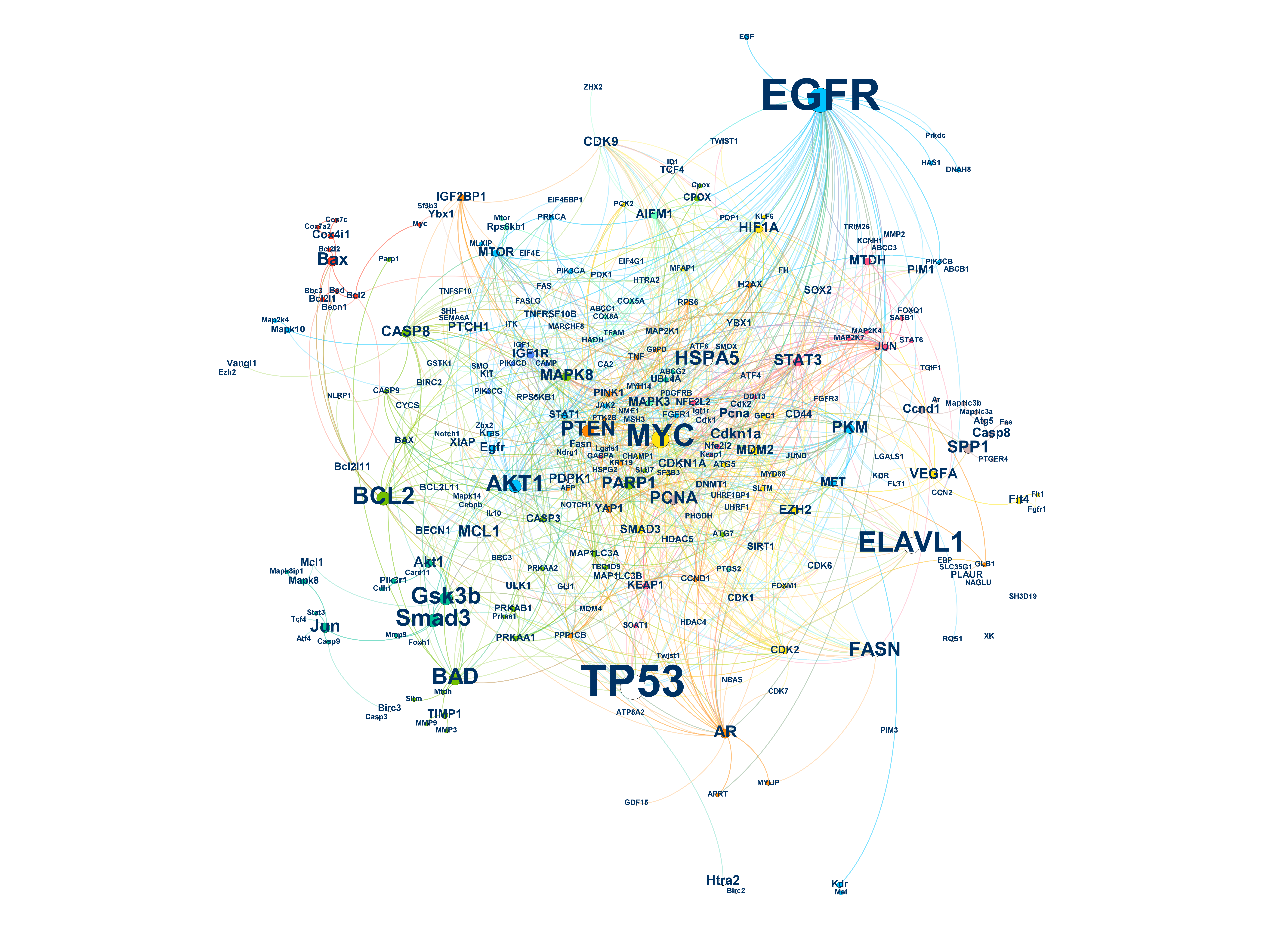
**

**Figure S5 Liver cancer network.** Each node represents a gene. The size of node indicates the importance of the gene in the network. The color of the nodes represents the network communities to which the genes belong. The edges between two nodes represent potential interactions between genes.


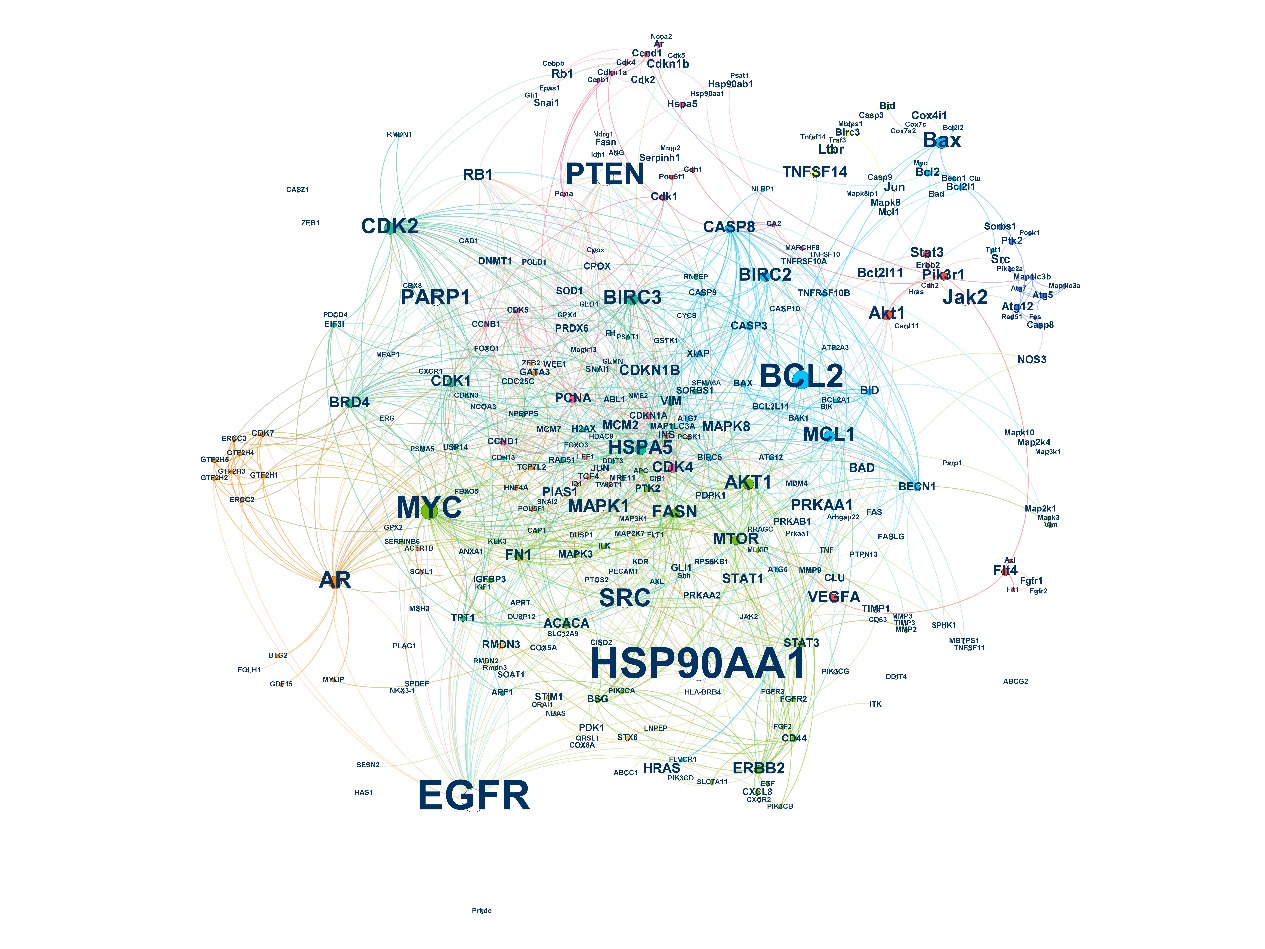


**Figure S6 Prostate cancer network.** Each node represents a gene. The size of node indicates the importance of the gene in the network. The color of the nodes represents the network communities to which the genes belong. The edges between two nodes represent potential interactions between genes.


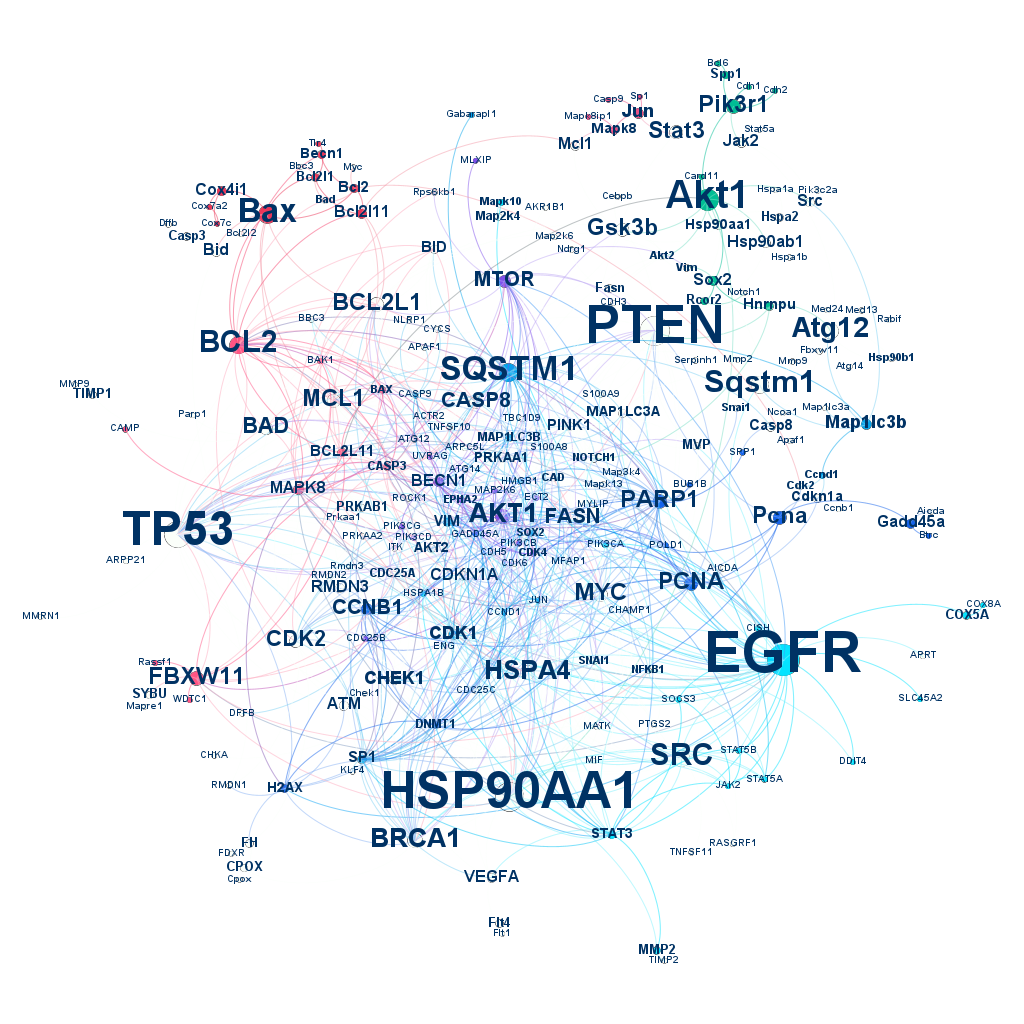


**Figure S7 Osteosarcoma cancer network.** Each node represents a gene. The size of node indicates the importance of the gene in the network. The color of the nodes represents the network communities to which the genes belong. The edges between two nodes represent potential interactions between genes.


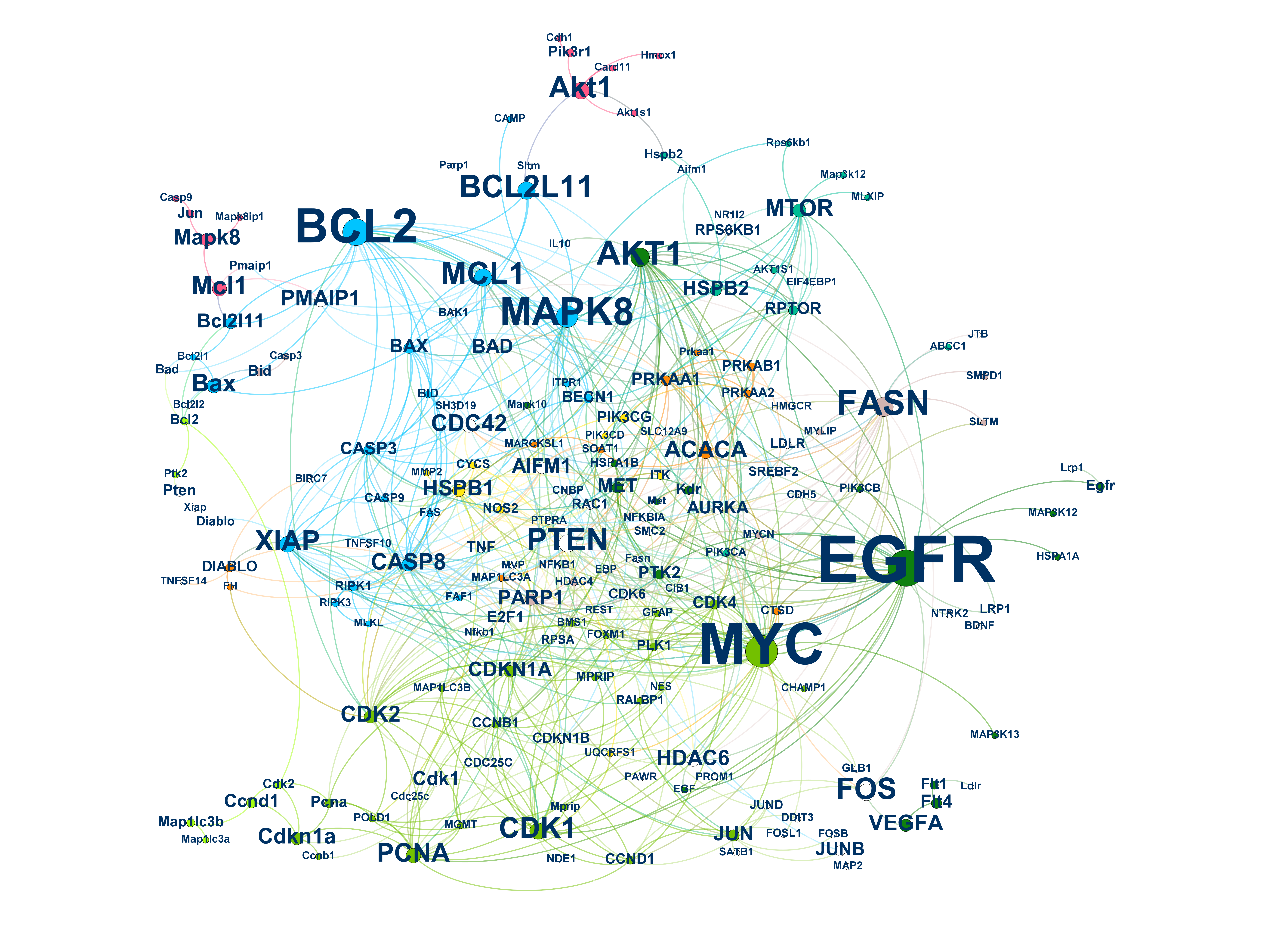


**Figure S8 Glioma cancer network.** Each node represents a gene. The size of node indicates the importance of the gene in the network. The color of the nodes represents the network communities to which the genes belong. The edges between two nodes represent potential interactions between genes.


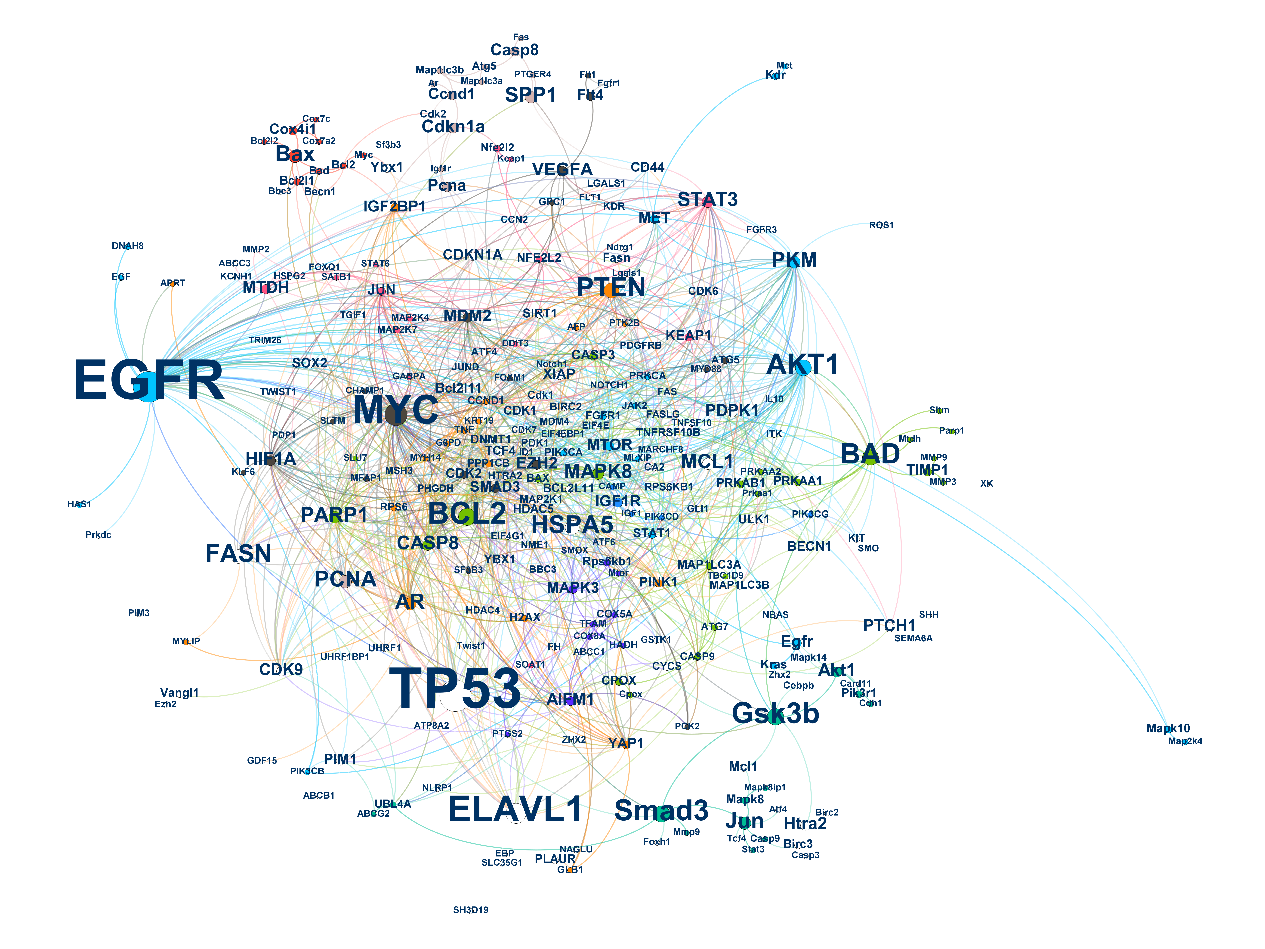


**Figure S9 Hepatocellular cancer network.** Each node represents a gene. The size of node indicates the importance of the gene in the network. The color of the nodes represents the network communities to which the genes belong. The edges between two nodes represent potential interactions between genes.

**
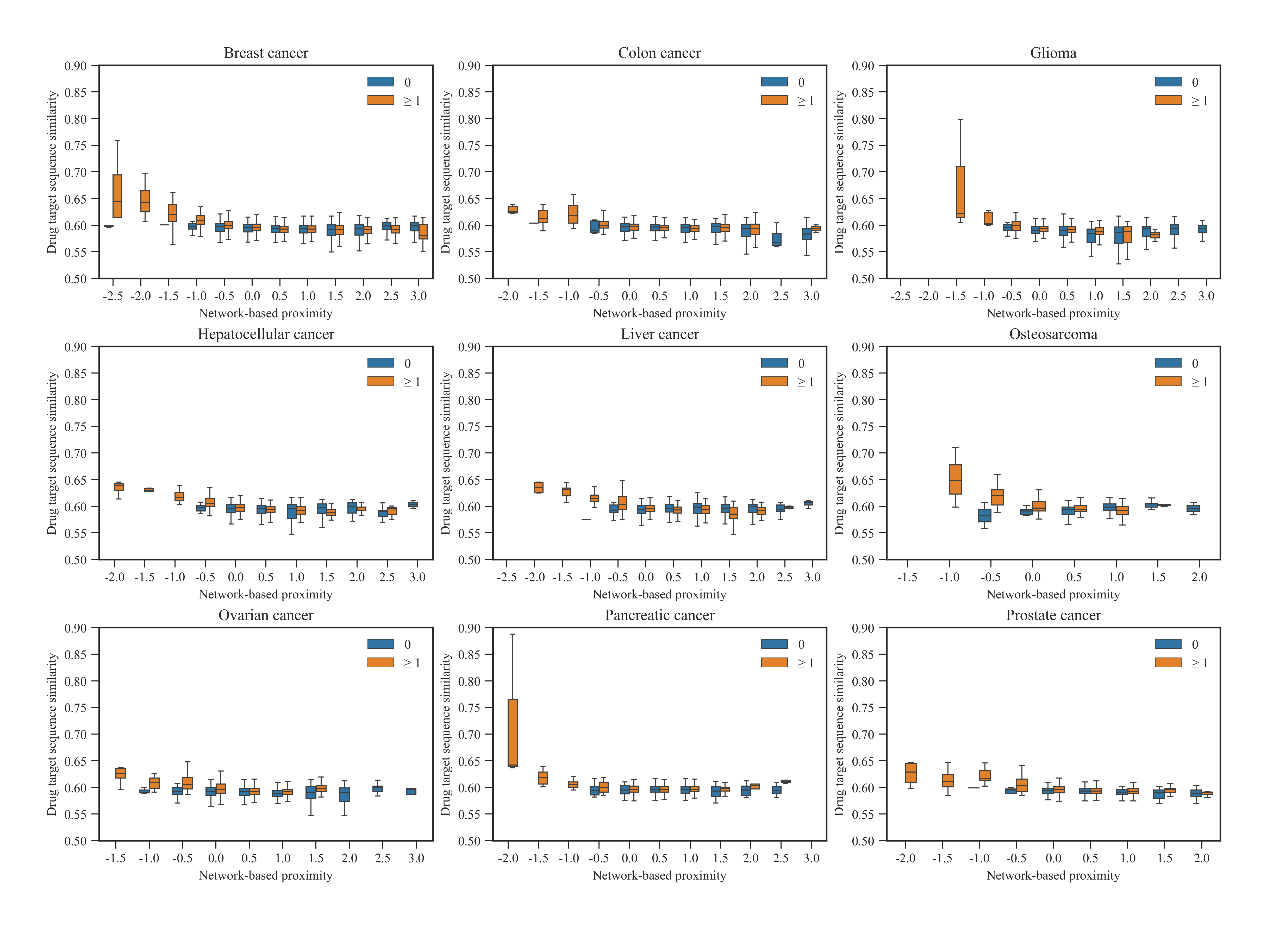
**

**Figure S10 Relationship between gene sequence similarity and network-based proximity in breast cancer, colon cancer, glioma, hepatocellular cancer, liver cancer, osteosarcoma, ovarian cancer, pancreatic cancer and prostate cancer.**

**
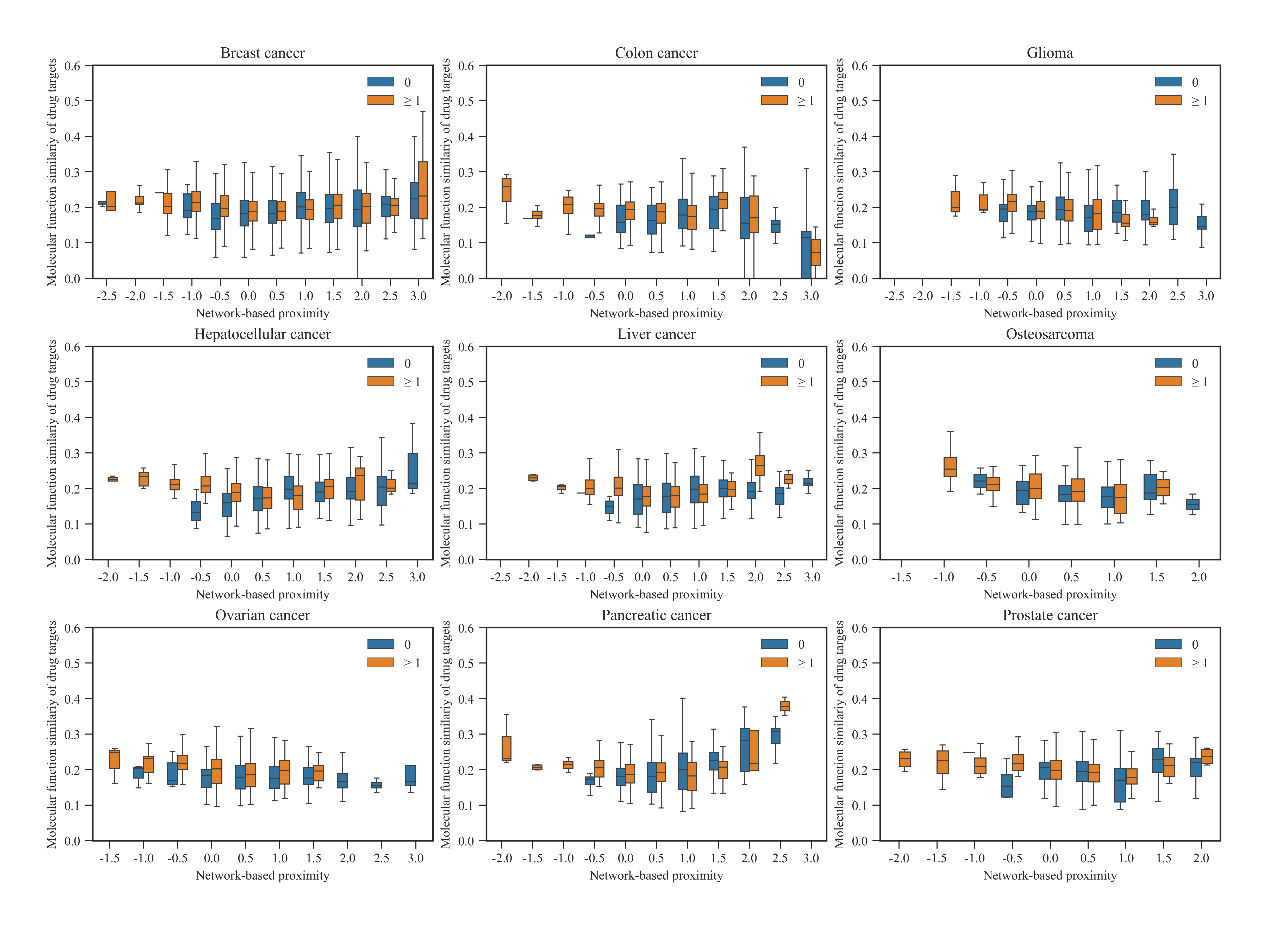
**

**Figure S11 Relationship between gene functional similarity and network-based proximity in breast cancer, colon cancer, glioma, hepatocellular cancer, liver cancer, osteosarcoma, ovarian cancer, pancreatic cancer and prostate cancer.**


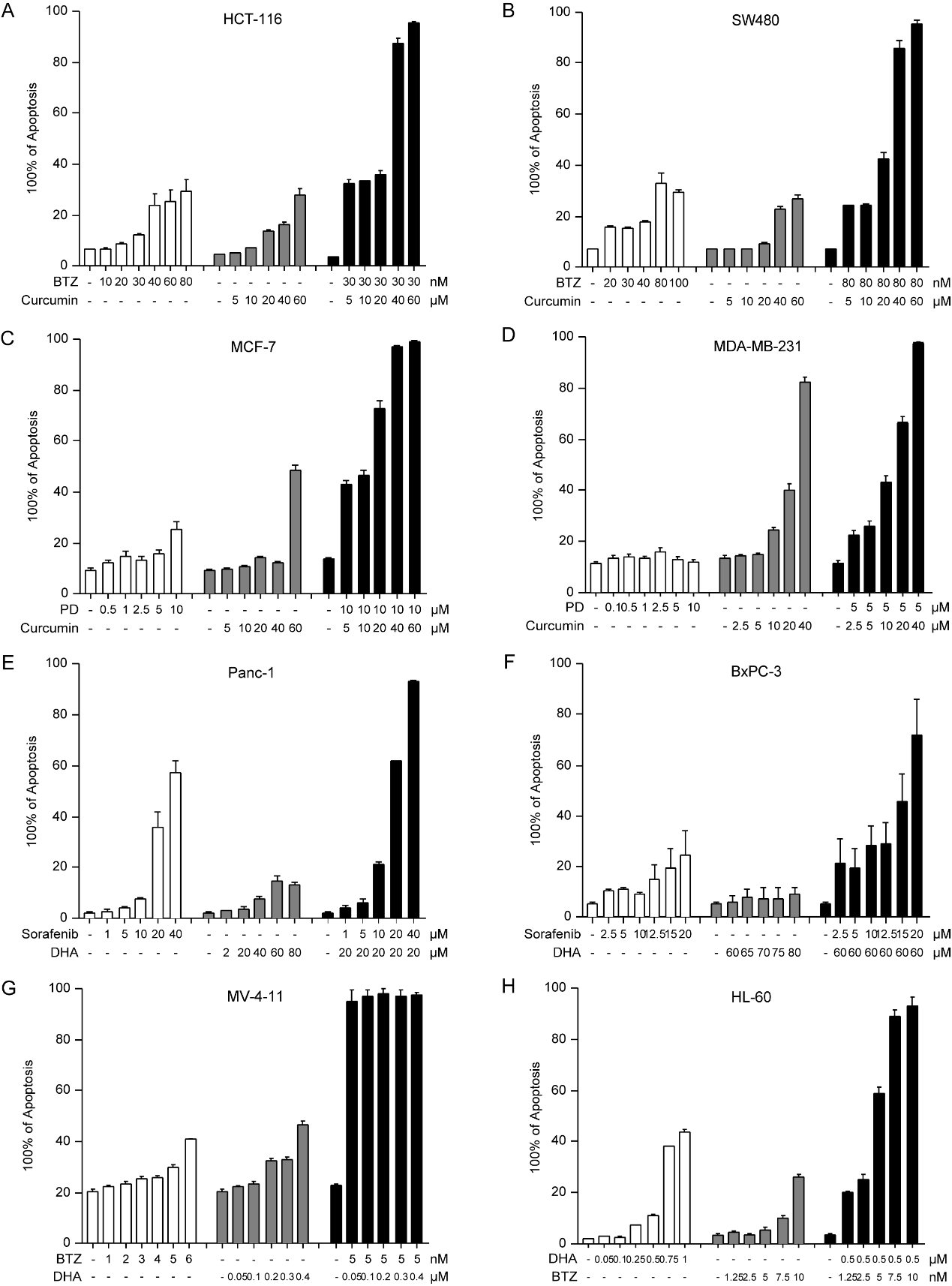


**Figure S12. Cytotoxic effects of various drug combinations in multiple cancer cells. A, B** Cell death of HCT116 and SW480 cells were assessed 48 h post treatment with indicated concentrations of BTZ, Curcumin, or BTZ/Curcumin combination. **C, D** Cell death of MCF-7 and MDA-MB-231 cells were assessed 48 h post treatment with indicated concentrations of PD, Curcumin, or PD/Curcumin combination. **E, F** Cell death of Panc-1 and BxPC-3 cells were assessed 48 h post treatment with indicated concentrations of BTZ, DHA, or BTZ/DHA combination. **G, H** Cell death of MV4-11 and HL-60 cells were assessed 48 h post treatment with indicated concentrations of Sorafenib, DHA, or Sorafenib/DHA combination. Data shown represent the means (±SEM) of biological triplicates.
